# Larger-scale ocean-atmospheric patterns drive synergistic variability and world-wide volatility of wheat yields

**DOI:** 10.1038/s41598-020-60848-z

**Published:** 2020-03-23

**Authors:** Ehsan Najafi, Indrani Pal, Reza Khanbilvardi

**Affiliations:** 1 Civil Engineering Department, The City College of New York, The City University of New York, New York City, 10031 USA; 2 NOAA Center for Earth System Sciences and Remote Sensing Technologies, The City University of New York, New York City, 10031 USA; 3 The Earth Institute, Columbia University, New York City, 10025 USA; 4 Present Address: Advanced Science Research Center, The Graduate Center, The City University of New York, New York City, 10031 USA

**Keywords:** Environmental impact, Environmental impact

## Abstract

Diagnosing potential predictability of global crop yields in the near term is of utmost importance for ensuring food supply and preventing socio-economic consequences. Previous studies suggest that a substantial proportion of global wheat yield variability depends on local climate and larger-scale ocean-atmospheric patterns. The science is however at its infancy to address whether synergistic variability and volatility (major departure from the normal) of multi-national crop yields can be potentially predicted by larger-scale climate drivers. Here, using observed data on wheat yields for 85 producing countries and climate variability from 1961–2013, we diagnose that wheat yields vary synergistically across key producing nations and can also be concurrently volatile, as a function of shared larger-scale climate drivers. We use a statistical approach called robust Principal Component Analysis (rPCA), to decouple and quantify the leading modes (PC) of global wheat yield variability where the top four PCs explain nearly 33% of the total variance. Diagnostics of PC1 indicate previous year’s local Air Temperature variability being the primary influence and the tropical Pacific Ocean being the most dominating larger-scale climate stimulus. Results also demonstrate that world-wide yield volatility has become more common in the current most decades, associating with warmer northern Pacific and Atlantic oceans, leading mostly to global supply shortages. As the world warms and extreme weather events become more common, this diagnostic analysis provides convincing evidence that concurrent variability and world-wide volatility of wheat yields can potentially be predicted, which has major socio-economic and commercial importance at the global scale, underscoring the urgency of common options in managing climate risk.

## Introduction

Wheat accounts for around 20% of the calories that humans consume and as such is the leading source of plant protein. It is well-known that wheat productivity is sensitive to both natural climate variability and extreme weather^1–10^. As a result, extreme weather disasters such as heatwaves, droughts, floods, cold spells, and the co-occurrence of compound extremes (e.g. hot and dry spell events) have caused significant production losses^11–14^. The relationships between climate, wheat production variability and stability, and socioeconomic outcomes has received growing attention recently^7,10,14–17^. Separate lines of evidence indicate that weather extremes across the globe can occur concurrently, due to mutual larger-scale climate drivers^18–20^, and that such larger-scale drivers influence global and regional crop productivity^7,10,21–29^. While agricultural influence of climate is well-established^1–17,21–29^, a detailed account of the characteristics of synergistic multi-national variability and world-wide volatility of crop yields, whereby many countries undergo harmonizing influences of climate to thwart or facilitate wheat productivity, needs more attension^10^.

**Figure 1:**
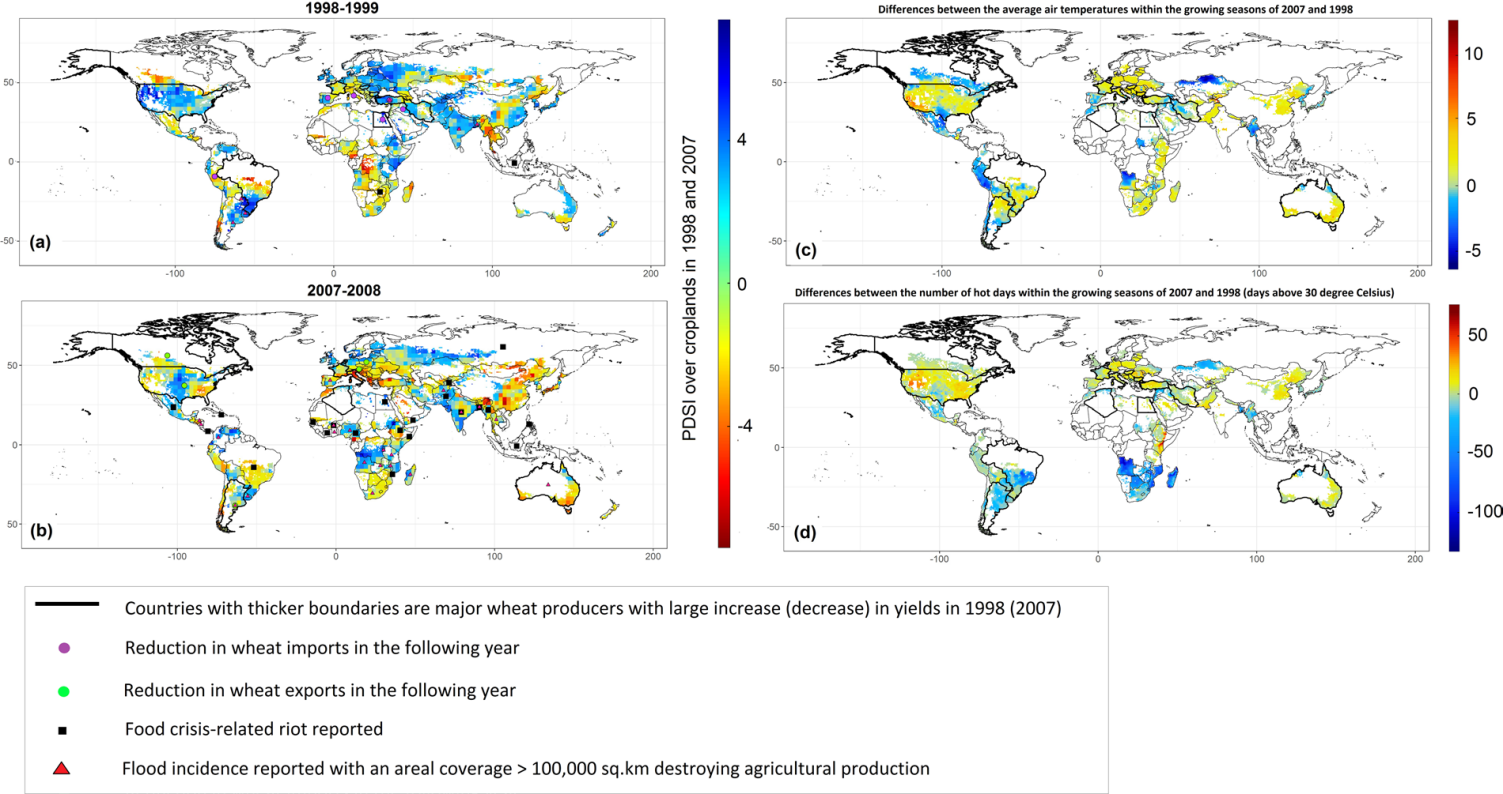
Maps highlighting countries (in thicker boundaries) where wheat yield anomalies (high or low) occurred across the globe, showing co-occurring local climate conditions and societal conflicts in years (**a**) 1998–1999 and (**b**) 2007–2008. Major producing countries having surplus yields (in 1998) or deficit yields (in 2007) are contoured in black colors. Countries having decreased imports (exports) in 1999 (2008) are designated by purple (green) circles in a (**b**). Reported food-related riots and/or crisis locations are marked by solid black squares (in a & b). Croplands experiencing extreme flooding incidence with more than 100,000 Km^2^ area are marked by red triangles (a & b). Colored grid points depict annual PDSI anomalies over croplands where negative (positive) indicating drier (wetter) conditions (a,b). (**c**) Map showing the differences in average local growing season Air Temperatures between 2007 and 1998. (**d**) Map showing the differences in the number of days within the growing seasons of 2007–08 and 1998–99 experiencing higher than 30-degree Celsius temperature. Please note that, a & b consider irrigated + rainfed (MIRCA2000)^68^ wheat growing fields while c & d locations showing areas where growing season information was available (SAGE)^49^. Food-related riots data was collected from the World Bank (https://www.worldbank.org/content/dam/Worldbank/document/Poverty%20documents/Introduction%20Guide%20for%20the%20Food%20Riot%20Radar.pdf.) (figure generated using R (http://www.R-project.org/) version R 3.4.2, ggplot2 (https://cran.r-project.org/web/packages/ggplot2/index.html) and Adobe Photoshop CS6 (https://www.adobe.com/products/photoshop.html).

History indicates that such synchronous volatility-led wheat yield losses can leave major ramification for global price and food security. Taken for instance, two crop-years: 1998–1999 and 2007–08 (Figs. 1 and S1). 1998–99 had one of the lowest and 2007–08 the highest global wheat prices in the recent history of the data (not shown), and 2007–08 was also one of the most yield volatile years characterized by the higher number of wheat-producing nations concurrently experiencing much below normal yields. On the other hand, 1999’s lowest price was notably accompanied by supply surpluses (Figs. 1 and S1), fewer number of droughts, fewer incidences of flooding events (red triangles), and closer to normal air temperature conditions over the key wheat growing croplands in 1998–1999 (Fig. 1). 2007–2008, instead, experienced producers largely reporting yield losses and supply shortages (Figs. 1 and S1), larger number of co-occurring extreme weather events such as droughts across Australia, eastern and southeastern Asia, and Europe, heatwaves in the USA, and floods in India and a range of African nations (Fig. 1). 2007–08, as a result, was one of the most yield volatile years in recent record (section SM1 in supplementary text provides more details on associated socio-economic implications).

Yet, we agree with Mehrabi & Ramankutti^7^, Anderson *et al*.^10^ and Gutierrez^16^ that existing science is still at its infancy to clearly diagnose and quantify the importance of systematic global-scale mechanisms (climate inclusive) by means of which multiple wheat producing nations “concurrently” encountered similar or opposite responses in wheat yields, leaving substantial impacts on modern-day international markets or synchronized global crop failure in the history^10^. Larger-scale climate drivers have the ability to exemplify variability of multiple regional crop productivity^10,21–24^, as they influence the world-wide climates^18–20^. The most popular ones include El-Nino Southern Oscillation (ENSO) cycles, Indian Ocean Dipole (IOD), North Atlantic Oscillation (NAO), and Scandinavian Pattern (SCA). Still, in what way one or more of such drivers influence a specific mode of world-wide yield variability or volatility characteristics in multiple nations, needs further attention from recent discoveries^7,10^. Hence, it is crucial to (i) decouple and quantify the leading modes of global yield variability, (ii) diagnose each principal mode based on country-specific local as well as larger-scale climate drivers, facilitating potential predictability assessment, (iii) investigate possible climate connections to world-wide yield-volatility trends - which sets the basis for this study.

## Results

### Leading modes of world-wide wheat yield variability

**Figure 2:**
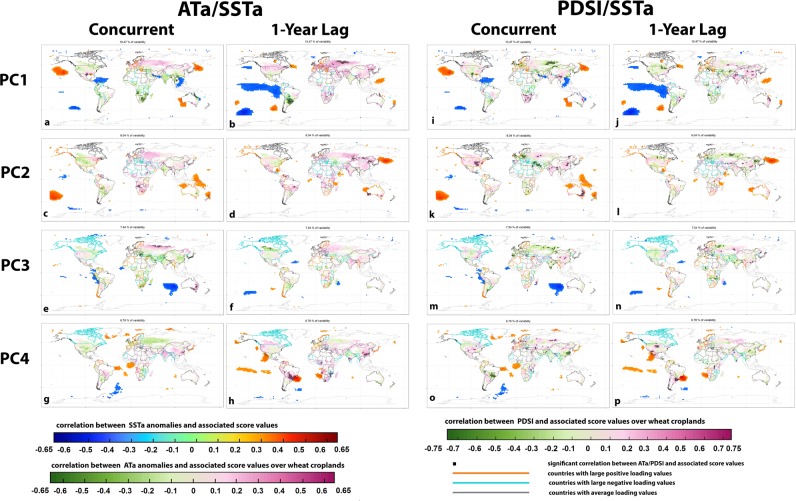
The concurrent year (and one-year-lagged) Spearman rank correlations between each PC time series, and climate variability. Palmar Drought Severity Index (PDSI) and Air Temperature anomalies (ATa) indicate local climate variability while Sea Surface Temperature anomalies (SSTa) indicate global ocean-atmospheric patterns. The locations with statistically significant correlations at the 95% levels are designated as small black dots over wheat cropland areas while the same over the global oceans are indicated by colors. The orange and blue colored country boundaries indicate nations with high concurrent variability in yields that is designated by high loading values within corresponding PC (Fig. S6). We label them as PCon# where # is the PC number (Table S1). It is important to note here that we kept all the regions growing irrigated and rainfed wheat for local climate analysis, irrespective of many those didn’t make it to our list of 85 countries considered in yield analysis (SM2 & SM14). (figure generated using R (http://www.R-project.org/) version R 3.4.2, ggplot2 (https://cran.r-project.org/web/packages/ggplot2/index.html) and Adobe Photoshop CS6 (https://www.adobe.com/products/photoshop.html).

Using standardized global yield data for eighty-five countries (section SM2 in supplementary text) and rPCA method^30^ (section SM3 and Fig. S2) we decoupled and quantified unique modes of world-wide yield variability in every principal component (PC) (Fig. S3). Those eighty-five wheat-producing countries account for nearly 83% of total global wheat production (according to 2013 statistics), but, the top ten PCs explained ~67% variance of global yields and the first four 33% (Fig. S3). Each PC implied a specific synergistic variability pattern in global yields wherein several producing nations contributed jointly in a variety of proportions (more in section SM4 in supplementary text). Figures 2 and S4 marked only those countries by thick orange/blue borders, which had high loading values (histograms of all loading values corresponding to 85 countries are shown in Fig. S6 and country names are in Table S1), designating countries participating in synergistic variability in yields. Countries corresponding to PC1 group (PCon1), included Nepal, Syria, DR Congo, Kenya, Niger, Tanzania, Tunisia, Austria, Bulgaria, Denmark, France, Germany, Greece, Hungary, Portugal, Romania, Sweden, Switzerland, Bolivia, Ecuador, Paraguay, Venezuela and New Caledonia, (Table S1, Fig. 2), several of which are major producing-, exporting-, and importing-nations (marked in Table S1). Section SM5 in supplementary text provides additional details of the countries exerting synergistic variability in global yields within PC2-4.

Spearman rank correlations between each PC and national-level yield variability provided indication on the extent to which each PC elucidated a national yield variance or vice versa (Table S2 and Fig. S7). We found that, while PC1 captured only about 10.5% year-on-year variance in world-wide wheat yields, it explained up to 74% variance in national yields (e.g. Romania was among the highest at 74% variance i.e. rank correlation equal to 0.86 and Syria was the lowest at 4% variance or rank correlation equal to 0.20, Table S2). Likewise, PC2 captured nearly 8% year-on-year variability in global yields, but explaining up to 58% variance in national yields (Australia was the highest at 58% or rank correlation equal to 0.76). Similarly, PC3 captured closer to 7.5% variance in global yields, but explained up to 55% national yield variance (rank correlation 0.74), and PC4 captured about 7% year-to-year variability in global yields, but explained up to 51% national yield variance (rank correlation 0.72). We have provided more details on all participating countries in Table S2 and Fig. S7.

Altogether, 33% global yield variability was captured by the first four PCs but each PC explained a much higher proportion of variance in individual national yields, providing confidence in potential predictability of multiple national wheat yields at a time.

### Local and larger-scale climate drivers provide diagnostic evidence of potential predictability of the leading PCs

Spearman rank correlation analysis between each PC and climate variability (data discussed in sections SM6-10 in supplementary text) indicated statistically significant influence of concurrent and previous-year’s climate drivers at the local and larger-scales. Within PC1 group of nations (Fig. 2, Table S1), Austria, Bulgaria, Denmark, France, Germany, Romania, and Paraguay were the leading producer and/or -exporter and/or -importers (method to choose country ranks is discussed in section SM11) whose wheat growing croplands (data discussed in SM13-14) indicated significant correlations between air temperature anomaly (ATa) in the previous year and PC1 (Figs. 2, S8 and S9). Such lagged correlation patterns provide fairly good indication of potential predictability of PC1 and with that multi-national crop yield variability (Figs. S8 and S9). For the sake of convenience, we designate these countries as hPCon1 (Fig. S9), which are also marked by * in Table S1. Some croplands within hPCon1 were also influenced by concurrent-year Palmer Drought Severity Index (PDSI) variability, but to a lesser degree (indicating the secondary influence of moisture in Denmark, France, Germany, and Paraguay). Altogether, nearly 3.7 million ha of wheat growing croplands within hPCon1 indicated statistically significant influence of local climate variability on world-wide wheat yield variability (as the synergy was captured in PC1), which was about 22.3% of the total wheat cropland areas within PCon1 nations, while nearly 32.6% fell within hPCon1’s wheat growing croplands.

In a similar manner, within hPCon2, the total growing area indicating concurrent influence of local climate on world-wide yield variability captured in PC2 was about 5-fold to that of PC1, which was nearly 18.2 million ha or ~32.4% and 38.5% respectively of the total wheat growing area falling within PCon2 and hPCon2 nations. Concurrent-year-average PDSI was the most dominating climate factor and second to that was lagged-ATa (Figs. S8 and S9). The same for hPCon3 were concurrent-year-average ATa where a total of 3.9 million ha of lands showed statistically significant correlations between concurrent local climate variability and PC3 scores, which was about 6.1% and 6.8% respectively of the total wheat-growing croplands falling within PCon3 and hPCon3 nations. hPCon4 group, on the other hand, indicated various degrees of influence by the different climate indicators, such as, concurrent-year’s average ATa in France, concurrent-year average PDSI in Germany, previous- and concurrent-year’s average PDSI in Spain and India, previous year’s average PDSI in Canada, and both lagged-ATa and concurrent-year average PDSI in Japan. It was about 3.2 million ha of croplands within hPCon4 or 6.9% and 7.1% respectively of the total wheat growing areas within PCon4 and hPCon4 nations indicated some degree of local climate influence on PC4.

Taken all together and considering the overlapping areas, there was around 78 million ha or 47% of the wheat-growing croplands globally experiencing some degree of local climate influence on synergistic yield variability within the eighty-five producing countries, and within them, nearly 29 million ha falling within the leading producers/exporters/importers.

Statistically significant correlations between each PC and the global sea surface temperature anomalies (SSTa) indicated systematic response of global wheat yield variability to the larger-scale climate variability (Fig. 2). The robustness of these SST correlations, indicating larger-scale influence, was further substantiated by a series of parallel correlation analysis using a range of standard ocean-atmospheric indices (data in SM10). Results altogether indicated that, there were several clusters of regions over the global oceans showing statistically significant correlations with the PCs (Figs. 2 and S4). The most notable one was the equatorial Pacific Ocean displaying a prominent ENSO-type pattern corresponding to PC1, at one-year lag. For PC2 also, the SSTa cluster over the tropical western Pacific and eastern Indian Oceans, around the Maritime continents and adjacent to northern Australia was significant. Existing literature indicated that the tropical Pacific’s ENSO is a primary driver of global climate variability^18–20^, influencing several croplands across the world^10,21,22^; but using different approach and datasets our analysis confirms that the tropical Pacific SST can explain two leading modes of world-wide wheat yield variability and beyond. This finding was further substantiated by another set of findings shown in Table S3 indicating that ENSO indices, peaking mostly in winter (December-January-February), associate best with PC1 variability, and to an opposite manner with PC2. There was also minor level of associations between PC1 and North Atlantic Oscillation (NAO); PC2 with the Scandinavian Pattern (SCA); PC3 with the Western Pacific (WP) pattern; and PC4 with the Tropical Southern Atlantic (TSA) pattern. Arguably, other physical climate indicators exist that possibly links with individual regional yields, such as, Indian Ocean Dipole pattern (IOD)^10^ and Southern Annular Mode (SAM) for Australia^24^ but country-wise diagnostic analysis was outside the scope of our study.

### Characteristics of world-wide yield volatility

Most yield Volatile Years (MVY) and Least yield Volatile Years (LVY) (method to pick these years was discussed in SM12 in supplementary text, and Fig. S10) indicate the highest and the lowest incidence of world-wide wheat yield volatility (Tables S4 and S5). Five of the top ten MVYs occurred in the recent most decades (Fig. S11, Table S4) while the same period of time only observed two LVYs (Fig. S11, Table S5). Volatility refers to yield spikes in both direction, that can be positive or negative, but in this case, we discovered that, MVY, in general, are mostly associated with world-wide grain shortages while LVY surpluses. There were about 10% reduction in wheat production, on average, during MVY, from the preceding year, which almost entirely came from the top yield volatile nations (Table S4), leading largely to total losses of 483 million tons of cereals. 2007 was the second highest MVY in record (status shown in Fig. 1 also), only after 1977 (Table S4). Paraguay, Austria, Australia, Turkey, and Argentina were some of the prominent wheat producers demonstrating highest degrees of yield volatility in the history.

**Figure 3:**
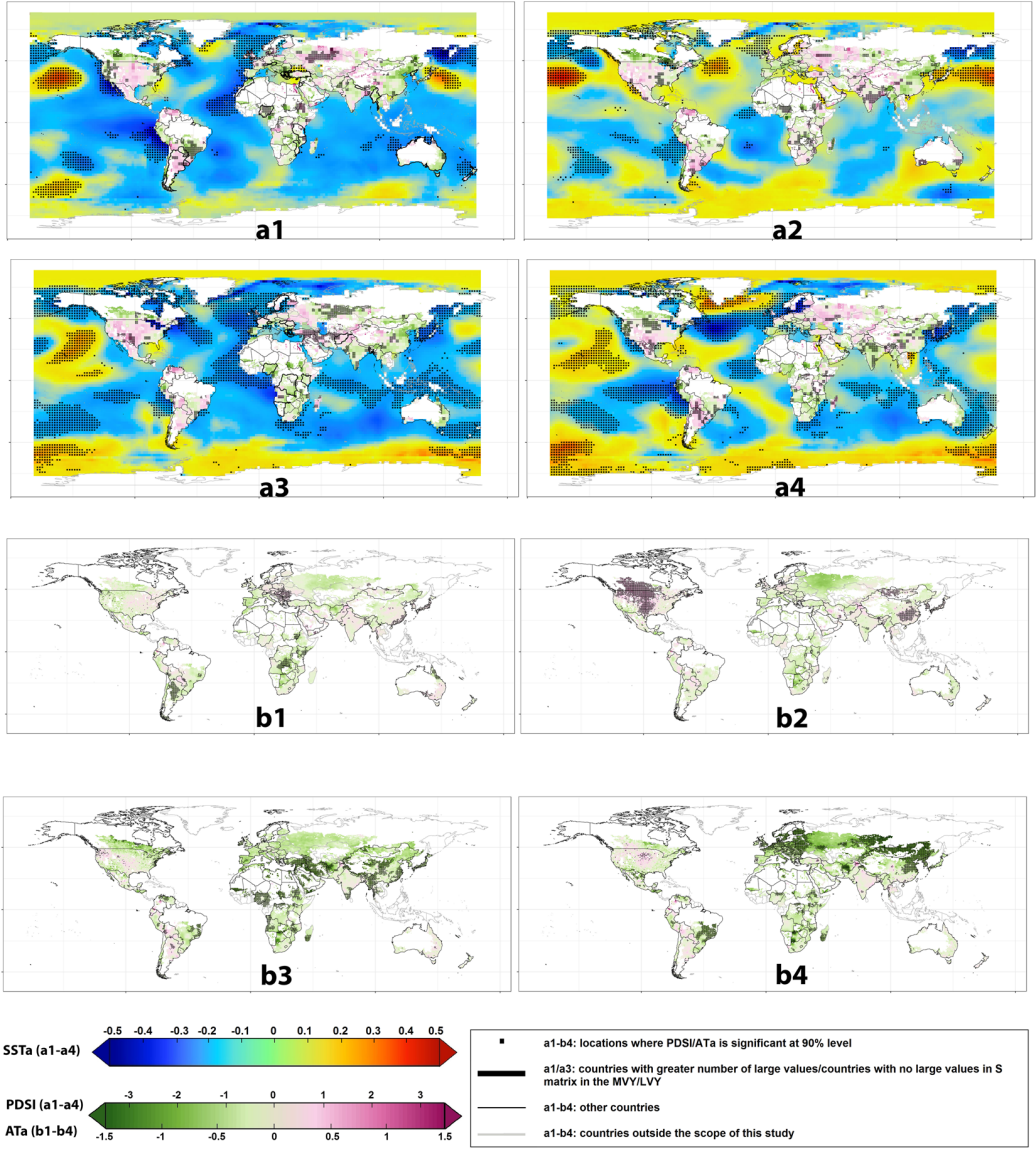
Composite maps of local and larger-scale climate drivers during the MVY and LVY years; (**a1**) SSTa and PDSI composites in the MVY years; (**a2**) the same in (**a1**) but at one-year lag; (**a3**) SSTa + PDSI composites in the LVY years; (**a4**) the same in (**a3**) but at one-year lag; (**b1**) ATa in MVY years; (**b2**) the same in (**b1**) but at one-year lag; (**b3**) ATa in LVY years; (**b4**) the same in (**b3**) but at one-year lag. The statistically significant locations over the croplands and oceans are marked by small black squares. Statistical significance was assessed by bootstrapping the anomalies (α = 0.1, 500 repetitions) at every grid cell. Countries with a greater number of large sparse yield values (#years) during the MVY/LVY, are represented by thicker boundaries in a1 and a3; Countries that are not in the scope of this study are highlighted as gray. Drier (wetter) conditions are indicated by large negative (positive) PDSI and hotter (cooler) conditions by large positive (negative) ATa. (figure generated using R (http://www.R-project.org/) version R 3.4.2, ggplot2 (https://cran.r-project.org/web/packages/ggplot2/index.html) and Adobe Photoshop CS6 (https://www.adobe.com/products/photoshop.html).

**Figure 4:**
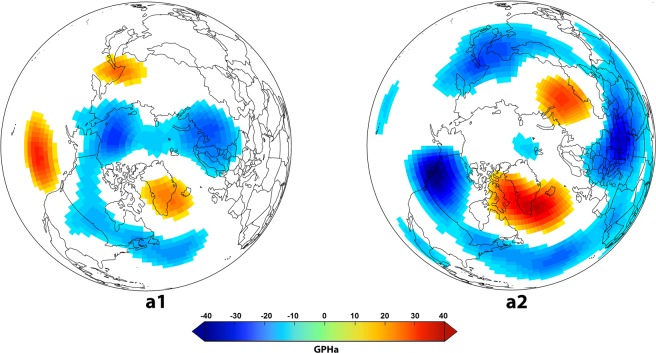
(**a1**) Geopotential Heights anomaly at 500hPa pressure level (Z500) in December, January and February (DJF) in the MVY over the North Hemisphere; (**a2**) the same in (**a1**) but in LVY. (figure generated using R (http://www.R-project.org/) version R 3.4.2, ggplot2 (https://cran.r-project.org/web/packages/ggplot2/index.html) and Adobe Photoshop CS6 (https://www.adobe.com/products/photoshop.html).

We further used a composite analysis approach, also called Superposed Epoch Analysis (SEA) (a method earlier used in the field^11^), to examine concurrent local and global climatic conditions during MVY and LVY (Figs. 3 and 4). The results were unforeseen and indicated a range of global patterns those were not found earlier in Fig. 2. These implied that larger-scale drivers associating with systematic world-wide wheat yield variability may differ from those inducing concurrent volatility. During MVY, in general, there were significantly higher number of extreme events happening all across the globe: for instance, concurrent drier and hotter conditions in Canada, the USA, Europe, and China; wetter conditions in Australia, western and northern Europe, India and Pakistan; and colder conditions in Australia, DR Congo, and Japan, among the most notable ones. As such, these leading producers- and/or exporters not only reported domestic wheat yield losses but also contributed to massive world-wide grain shortages.

Using the same SEA method, we further found that MVY followed years when the global oceans in the northern Hemisphere were warmer on average (Fig. 3-a2), along with a significant see-saw-like SSTa pattern over the northern Pacific and adjacent to Alaska (Fig. 3-a1), a warmer blob over northwestern Atlantic (Fig. 3-a2), and a NAO-like feature observed over the northern Atlantic (Fig. 4-a1). The PDSI composites in Fig. 3 a1 & a2also support expected teleconnections from the NAO-like feature, as one would expect, as wheat is primarily moisture limited in Spain and North Africa (-ve PDSI) and the NAO affect wheat yields in these regions by altering moisture availability^10^, while in central Europe and Scandinavia the temperature teleconnection is generally stronger (Fig. 3-b1,b2). During LVY, on the other hand, we observed a horseshoe-shaped patch of warmer water in the northern Pacific shifting its position towards the eastern side of the basin while a cooler patch almost disappearing from the north-eastern Pacific (adjacent to the western USA) going from MVY to LVY (Fig. 3-a3, a4). The global oceans followed a much cooler trend during LVY with the most notable cooling feature occurring over the northern Atlantic (both during concurrent year and previous year) (Fig. 3-a3,a4). A cooler central and eastern Pacific Ocean (ENSO region) was significant during LVY (Fig. 3-a4), (adjacent to South America). Hence, it was most interesting to note that while ENSO explained primary modes of variability in world-wide wheat yields (Fig. 2), it became much lesser of significance for the most volatile years and yield losses across the world. Rather, a La-Nina type feature showed up during least volatile years. A direct composite analysis with the same indices as in Table S3 also confirmed that there was no significant feature of the El-Nino cycle co-developing within MVY. Instead, there were other ocean-atmospheric patterns indicating co-occurrence over the global oceans during the MVY and LVY. Examples of those patterns included negative winter-time NAO (−0.34), negative SCP (−0.28), and positive SAM (0.27) co-occurring with MVY; while winter-time negative PNA, and negative Quasi-Biennial Oscillation patterns (−2.2) co-occurring during LVY, but these findings are preliminary and hence warrant a more in-depth analysis for confirming physical significance^10^.

### Comparison with existing literature

Previous studies suggested that wheat yields depend on local climate variability^6,11^, and are influenced by larger-scale ocean-atmospheric patterns^10,21,22^. The recent research is however gradually addressing^7,10^ (our study inclusive), how and to what extent multiple national wheat yields vary synergistically^10^ and be concurrently volatile^7^ where local and larger-scale climate dynamically play a central role. Nevertheless, many of our diagnostic outcomes well corroborate with existing research as it also takes existing knowledge forward, which we discussed below:

We found that global wheat yield variability can be decoupled into and quantified by different unique modes, where, ~33% can be captured by the first four PCs (Fig. 2, the first 10 PCs account for ~67% of the total global yield variability. PC5 to PC10 are exhibited in Fig. S4). Each PC indicated a synergistic variability pattern in multiple national yields where a range of leading producers participated concurrently. These joint variability patterns in multi-national wheat yields could be simultaneously explained by specific local as well as larger-scale climate drivers. Previous year’s air temperature anomalies recorded over the wheat croplands as well as ENSO-type characteristics across the tropical Pacific Ocean indicated dominating influence on PC1, and to some extent on PC2 also. Concurrent-year-average PDSI and previous year’s air temperature variability was important for PC2, while concurrent-year air temperature for PC3, and a mixture of air temperature and precipitation variability for PC4 depending on the country of interest. Among all the recent literature we reviewed, Ray *et al*.^6^ was most comprehensive and recent global assessment to our knowledge, that used a sub-national yield dataset at the annual scales, and examined influence of local temperature and precipitation variability on region-specific wheat yield variations. They indicated that climate variability explained up to 35% of global wheat yield variability where temperature was the leading factor for wheat yields, in general, across the world. Adding on to Ray *et al*.^6^ and other relevant local studies^31,32^, our findings revealed that annual wheat yields concurrently vary across many nations but by a variety of proportions. One set of country examples is: Nepal, Syria, DR Congo, Kenya, Niger, Tanzania, Tunisia, Austria, Bulgaria, Denmark, France, Germany, Greece, Hungary, Portugal, Romania, Sweden, Switzerland, New Caledonia, Bolivia, Ecuador, Paraguay and Venezuela (Table S1, Fig. 2) where synergistic yield variability patterns were captured in PC1 (others are in Table S2). It was also exhibited in Ray *et al*. and others^6,31,32^ that temperature variability in Western Europe is more crucial for wheat yields than precipitation variability, where the only exception was Spain where precipitation variability was important. Now, referring back to Table S1, our analysis also discovered that yield variability in Spain fell under PC4 category showing PDSI variability as the sole important factor. Broadening this discussion, Ray *et al*.^6^ also examined Australian wheat yields, which is mostly rainfed^33,34^, and hence it made sense that it would be largely explained by precipitation variability. We too denoted that, PC2 not only explained Australian yield variability at largest but also indicated PDSI variability being the most crucial local-scale climate driver. Furthermore, ATa at 1-year lag indicated second important factor for PC2. This is important as the planting and growing season of Australia and other countries in PC2 list starts in the previous year (crop years are in Table S6). United States, Turkey, and Iran also indicated concurrent yield variability with Australia within PC2, where PDSI variability (wet/dryness) during the concurrent year was also the most dominating factor and ATa the second. India was another good example to mention here, where our study once again corroborated well with Ray *et al*.^6^, indicating previous- and concurrent-year’s precipitation variability i.e. moisture being important within PC4 category. In addition, ENSO has much smaller influence on wheat production of India (Rabi crops is winter wheat) and China^10^, because wheat is heavily irrigated in these countries and hence irrigation explains the muted effect of climate modes on wheat.

Globally, 67% of the croplands are located in the areas where one or more climate oscillations show statistically significant alterations in crop productivity during their strong phases^21^. Adding to that knowledge, we presented that there were around 47% of wheat-growing croplands experiencing some degree of local climate influence within the eighty-five producing nations, falling especially within PC1-4 category. Our diagnostic results also provided indications that previous year’s tropical Pacific Ocean SSTa was the most dominating stimulus for world-wide wheat yields (PC1 and PC2), indicating that potential predictability in multi-national yields is possible and can be higher for PC1. We also found minor level of associations between PC1 and North Atlantic Oscillation (NAO); PC2 with the Scandinavian Pattern (SCA); PC3 with the Western Pacific (WP) pattern; and PC4 with the Tropical Southern Atlantic (TSA) pattern.

Knowledge of the regional connections between larger-scale climate and food crop production existed, but the global studies were really few^10,21,22^. Among those, the methods and the time spans were not fully consistent, which already in themselves caused differences in many of the results. In addition, many of these studies either examined crop-specific relationships, e.g.^22,35^ or studied aggregated yields of the major crop types^21^ or considered only one specific type of larger-scale driver^22^ or didn’t consider concurrent local climate statuses with larger-scale climate modes^10^. Heino *et al*.^21^ was the first global assessment, to our knowledge, which incorporated ENSO, IOD, and NAO altogether impacting 12 major crop types globally. They however took mean crop productivity at sub-national scales, not separating out crop-specific controls. Heino *et al*.^21^ indicated that 27% of global mean crop production is sensitive to ENSO variability, while 5% to IOD, and 20% to NAO, showcasing the dominance of ENSO on world-wide croplands. Anderson *et al*.^10^ was the most recent study reporting that ENSO, IOD, tropical Atlantic variability (TAV), and NAO together accounted for 6% of globally aggregated wheat production variability but ENSO had a substantial influence on global crop production. Along that line, we discovered that it is ~11% of world-wide variability in wheat yields, as captured within PC1, those could be largely explained by ENSO variability alone.

Heino *et al*.^21^ further demonstrated that aggregated crop productivity (12 types) was majorly insensitive to ENSO/IOD/NAO in Eastern Europe, Central Asia, North America, Western Europe and Central America. This was where our study particularly found some differences, because PC1 and PC2 of world-wide wheat yield variability included countries in Europe, Asia, and North America showing significant associations with ENSO cycles, in various degrees (please refer further to the discussion section). Our study also corroborated well with Iizumi *et al*.^22^ that was an earlier global assessment to^21^ but solely included ENSO in their study. Iizumi *et al*.^22^ showed that ENSO phases influence wheat yields in many parts of South Asia, Latin America, and Southern Africa, both positively and negatively. The more recent study by Anderson *et al*.^10^ indicated that a developing ENSO event in boreal summer forces wheat production anomalies in Eurasia and a decaying event in the following spring produces anomalies in Australia and southeast South America. FAO^36^ specified observed impacts of 2015/16 El-Nino on Latin America, Africa, and Asia. There were also other local case studies identifying ENSO’s impacts on crop production in the United States^10,37^, Zimbabwe^38^, Argentina^39^, China^40^, and Indonesia^35^. Among them, Anderson *et al*.^10^ also indicated Australian and East African crop yields as affected by IOD, Yuan and Yamagata^24^ indicated stronger influence of the IOD than ENSO on Australian winter wheat yields, and also specifying the possible influence of SAM, for the first time, on Australia’s climate and yields. Whereas, our findings provided a different level of evidence indicating that a fraction of Australia’s annual wheat yield variability was largely explained by PC2 and that is primarily influenced by ENSO in the concurrent year (Fig. 2) and to some extent by SAM (Table S2), and indicating no significant influence of IOD. This was perhaps because IOD influence is more “localized”, as Yuan and Yamagata^24^ also pointed out, and ENSO development stage (timing) is crucial for a country’s crop production where a decaying stage in the spring is supposedly crucial for Australia^10^.

## Discussions

The importance of climate drivers arises from the influence on communities, businesses, and socio-economy^41^. Diagnosing the predictability of these effects in the observed data can help to anticipate future outcomes. Wheat is one of the principal cereal crops grown worldwide. Consumers in many countries are increasingly dependent on food imports and are thus exposed to yield variability in the major food-producing regions^42^. National governments and commercial entities are therefore paying particular attention to the climate risk of wheat important for exporting as well as importing countries at large. Given the rising incidence of climatic extremes affecting food production^11,43,44^ it is important to anticipate larger volatility in food productivity^45,46^. Global crop models and yield monitoring (such as, the Global Information and Early Warning System of the Food and Agriculture Organization of the United Nations and the Famine Early Warning Systems Network) have been developed^47,48^, but, to our knowledge, only a few research yet have evaluated the common diagnostics and predictability potential of multi-national yields^10^, but neither looked into the changing characteristics of world-wide yield volatility. Here, we conducted a global overview of the concurrent variability and volatility of wheat crop yields using national-scale wheat yield datasets from 1961–2013 and climate variability, where ocean-atmospheric processes have often produced simultaneous global influence and altered climates around the crucial croplands from one year to the next.

Our research presented three major aspects for the science and broader readership. First, it provided insights on the synergistic variability of and diagnostics for multi-national wheat yields observed across the world. Second, it revealed a statistically significant and physically meaningful response of multi-national yields to larger-scale climate drivers at longer-range time scales, providing predictability potential. Third, it presented changes in global yield volatility characteristics.

First: We characterized concurrent variability of wheat yields using the rPCA approach which quantified unique variability patterns of world-wide yields into PCs and showed where and by how much, multiple national yields concurrently varied. The top four PCs demonstrated nearly 33% of the global yield variance where a total of 23 producing nations had dominating influence on PC1 (very high loading values), indicating substantial co-variability of yields. Among them were the top producers such as Austria, Bulgaria, Denmark, France, Germany and Romania in Europe and Paraguay in South America where local climate influence was significant, indicating a dominance of air temperature variability at one-year lag period and concurrent-year PDSI variability as the secondary influence. This echoed previous findings, especially of Ray *et al*.^6^ and Iizumi *et al*.^22^, which we discussed in the earlier section. Although more wheat croplands across the globe are rainfed than irrigated, dominance of temperature influence than moisture is particularly true in irrigated areas also where yields might be sensitive to temperature as it is a major driver of yield variability if a crop is sufficiently irrigated, whereas the soil moisture content can be more important under insufficient irrigation conditions^22^.

The importance of air temperature being the most important factor makes sense as it drives a farmer’s decision on wheat planting dates, everywhere across the world^49^. Winter wheat in the northern mid-latitudes is mostly planted in autumn (September and October, in general) when temperature is <4 °C) as winter wheat requires colder winter temperature for vernalization. Winter wheat begins to grow before the winter sets in, becomes dormant during the winter and then resumes growth in the following spring. Therefore, farmers choose a winter wheat planting date, according to temperature variability during the planting month, to also ensure that a proper amount of growth of the crop is achieved before winter dormancy sets in. As such, growing degree day accumulation between planting date and the onset of cold temperature is an important climatic factor no matter how variable that is between different regions as the temperature at planting varies between regions. Austria, Bulgaria, Denmark, France, Germany, Romania, and Paraguay (hPCon1) grow winter wheat (Table S6). Farmers in these regions might be choosing planting dates by favorable temperature conditions, ensuring a critical growth stage of winter wheat in the following year, such as flowering. Hence, previous year’s air temperature’s importance for PC1 for all these regions make sense to induce synergistic yield variability recorded in the following year (for further information about other countries and PCs, e.g. Australia, whose previous year’s climate indicators also showed importance in PC2 for the reason that planting and growing season falls in the previous year and then continues into the next year, please refer to Table S6). As such, farmers can better strategize in these countries to adjust their planting dates according to climate forecast information, to maintain or increase crop yields in the face of increasing climate variability and global climate change impacts. In addition to that, global crop models assume relationships between climate and planting dates too to simulate crop yields. Therefore, our research solidifies the importance to design multi-national strategies for climate adaptation.

We also indicated the critical importance of previous year’s ENSO on PC1, whereby European growers and Paraguay are most influenced and thereby their yield variations. This can be explained by the known influence on the extratropical winter atmosphere by events in the tropical regions^50^. Different climate studies have indicated that temperature and precipitation variability across North Atlantic/European regions and South America follow patterns consistent with ENSO-related larger-scale climate dynamics^51–55^, influencing crop productivity in these regions^21,56^. Anderson *et al*.^10^ explained that, in Europe, both ENSO and the NAO affect winter wheat yields, but ENSO does so by forcing NAO-like atmospheric states in the North Atlantic. This partly explains our findings relating to persistent ENSO associations with PC1 and its correlation with previous year’s air temperature variability in Europe and Paraguay. Given the incredible level of ENSO predictability already achieved in seasonal forecasts, these climate diagnostics can offer the opportunity of predictability for PC1 (and PC2 to some extent) and hence co-varying yields in multiple nations during active ENSO years as well as assessing the risk of future changes.

Next: We also studied global yield volatility characteristics. We noted that extreme yield volatile years have become common during the recent most decades when the world has lost more on yields than gains. Five of the top ten such extreme years have occurred between 1990–2010 as the oceans also got warmer in the northern hemisphere, indicating the impacts of climate change. Previous studies have indicated that low-yield variability potentially leads to^57–59^ higher food supply^60,61^, and prevent price spikes that have disproportionate adverse impacts on the globally food-insecure who are mostly farmers^62,63^. Ray *et al*.^6^ indicated that annual yield variability can be generally lower in the top crop producing regions due to higher yields but the major exception can be Australian wheat belt and the Great Plains states of the United States. Therefore, our findings set a new alarm by introducing many other prominent wheat growers along with Australia, including countries such as Paraguay, Austria, Turkey, and Argentina showing increasing volatility in yields and inducing declining world-wide wheat productivity. Regions with higher crop yield volatility can as such lead to disproportionate productivity failure and global food price spikes, especially if they are the major breadbaskets of the world. Even in regions with comparatively lower yields, fluctuations in crop production may impact local food security and farmers’ livelihoods. Our study therefore provides a global picture but keeping a local view intact of regions those could continue to threaten the wheat system at different scales.

Taken all the discussions above, our study is distinct compared to previous investigations that explored yield variability^2,6,10,14,15,25,26^ but without characterizing concurrent volatility in wheat yields together. We filled that crucial gap, using rPCA method that differs from traditional PCA as it removes existing outliers from the standardized crop yield datasets that would otherwise impact the PC outputs and thus is more “robust” (section SM3). In addition, we argue that rPCA may also have the ability to permit the interplay of important climatic determinants as well as other socioeconomic factors^64^, such as fertilizer prices and oil prices, thereby disentangling global climatic teleconnections from other possible sources in PCs. While these global non-climatic drivers were not within the scope of our study, they may appear in other PCs.

Another original contribution of our work was the analysis of outliers, which our results suggested, can be useful to characterizing global yield volatility that may (or not) emerge from persistent yield variability patterns^17^, and mainly leading to world-wide yield losses and potential food insecurity. This strategy allows us to consider climatic inter-connections across the worlds’ wheat growing regions, and as such, we showed that it is not only international yields that can co-vary, largely due to climatic reasons, but extreme volatility can also appear in association with climatic teleconnection patterns, where the reasons of volatility may not be due to the same larger-scale influence corresponding to persistent variability.

Extreme weather can cause local crop failure and negatively affect socio-economies around the world, with implications for global market price and societal conflicts (Fig. 1). We showed, using significant association mapping, that, modes of international yield variability associates with larger-scale patterns, but, warmer global oceans associate best with extreme yield volatility-led losses across the globe. As such, multiple lines of evidence, including correlation and composite analysis support the hypothesis that multiple wheat-producing nations respond concurrently to larger-scale climate drivers, where, the most serious impacts occur in both big and small wheat-producing nations. Our observation that such synchronous volatility-led yield losses have become more frequent in the recent two decades is concerning and has implications for global food security planning and management. Given that wheat is a heavily traded commodity in the global market (http://www.fao.org/faostat/en/#data) and that some of the most productive (and/or food insecure) regions across the globe observe higher degree of yield volatility as oceans warm^65,66^ and extremes become more extreme^44,67^, our study takes a meaningful step forward, from existing knowledge, by discovering the common mechanisms by which multiple vulnerable nations can manage disastrous extreme climate events and yield responses and minimize significant impacts on international crop markets, society of the food insecure nations, and farmers at large.

## Methods

All the datasets used, and methods considered to analyze them are included in Supplementary Text and cited in appropriate locations within the manuscript. The data, along with methods included in the codes will be made publicly available on a publicly accessible repository (https://datadb.noaacrest.org/public/gfsc-gws/).

## Supplementary information

Supplementary information.

## Data Availability

All the data, along with codes written to produce figures are made publicly available on a publicly accessible repository (https://datadb.noaacrest.org/public/gfsc-gws/).
