# Supplementary material for: Larger-scale ocean-atmospheric patterns drive synergistic variability and world-wide volatility of wheat yields
Source: Sci Rep. 2020 Mar 23;10:5193. doi: 10.1038/s41598-020-60848-z (PMC7090071; doi:10.1038/s41598-020-60848-z)

Supplementary Materials

for

Larger-scale ocean-atmospheric patterns drive synergistic variability and world-wide volatility of wheat yields

Ehsan Najafi[1], Indrani Pal[2,3,*], Reza Khanbilvardi[1,2]

[1]*Civil Engineering Department, The City College of New York, The City University of New York, New York City, USA, 10031*

[2]*NOAA Center for Earth System Sciences and Remote Sensing Technologies (NOAA-CREST), The City University of New York, New York City, USA, 10031*

[3]*Columbia Water Center, The Earth Institute, Columbia University, New York City, USA, 10025*

[*]*Corresponding author: Indrani Pal, ipal@ccny.cuny.edu / ip2235@columbia.edu*

Supplementary Text

SM1. Socio-economic implications of lower and higher world-wide yield volatility in 1998-99 and 2007-08: Figure 1(a) indicated that, in 1998-1999, global yield volatility (measured by the number of nations having standardized yields departing by more than one standard deviation value from the global normal) was relatively lower when higher number of producers recorded yield surpluses (Figure S1). Major importers, such as, Egypt, Italy, Spain, Iraq, Turkey, and Peru, reduced their annual wheat imports due to surplus domestic production. There were significantly fewer food-related riots all across the globe, and a plunge in global market price of wheat in 1999, which was one of the lowest in the recent history. In 2007-2008, as in Figure 1(b), major wheat-producing countries experienced greater degree of yield volatility and concurrent yield losses (Figure S1). Major exporters such as the USA, Canada, Australia, Argentina, among others, reduced grain exports. As a result, Argentina imposed higher tariffs on grain exports, and the USA and Germany imported additional grains to meet domestic demand. Many crucial importers such as Algeria, Morocco, Iraq, and Turkey relied heavily on imported grains to meet demand. 2007-2008 reportedly experienced significantly higher number of food-related conflicts across the globe and the global market price reached record high in 2008.

SM2. Wheat yield data and standardization method: Wheat is grown on more land area than any other food crops (220.11 million hectares, according to FAOSTAT, http://www.fao.org/faostat/en/#data/QC). Only after sugar cane and maize, wheat had the largest production recorded in 2016 (~749.46 million tons). We used wheat yield dataset downloaded from FAOSTAT for all the producing countries across the world but selected those having a complete year-on-year record from 1961-2013 (http://www.fao.org/faostat/en/#data) or a minimum number of repeating yield records in the consecutive years.

Due to technological improvements over time, each yield time series was expected to have a monotonic trend component. We were primarily interested in persistent yield variation and therefore we de-trended each time series using a standardization approach adopted in earlier research[1] (Equation 1).

$Y_t = [y_t – \text{mean}(y_{t-3,t+3})] / \text{SD}(y_{t-3,t+3})$ ……………………………Equation 1

In Eq. (1), Y_t is the detrended yield value at year t, y_t is the original yield value at year t, $\text{mean}(y_{t-3,t+3})$ is the mean of the original yield values for the seven-year moving window centered around year t, and $\text{SD}(y_{t-3,t+3})$ is the standard deviation of the original yield values for the seven-year window centered around year t. The final yield dataset contained standardized wheat yield anomalies for 85 countries from 1964-2010 where 1964 contains the yield value of that year standardized with respect to the annual yields recorded within 1961-1967, and so on. These 85 countries accounted for about 83% of global wheat production (as per 2013 statistics). It should be noted throughout within the manuscript that all the reported yield numbers are based on 85 countries studied and missing countries include some of the major wheat producers such as Russia, Ukraine and Kazakhstan. As such, global wheat cropland area refers to cropland areas of wheat within the 85 countries. More discrepancy in the data used is mentioned in SM14.

SM3. Robust Principal Component Analysis (rPCA) method[2]: When a dataset, such as global crop yields (85 countries and 47 years), has such a higher dimension, reducing its feature is crucial[3] to meeting our overarching goal. Principal Component Analysis (PCA) is a useful approach for dimensionality reduction in earth sciences that enables better diagnostics of the structure of the global data at multi-dimensional space and time[4]. When there are outliers in a

sample dataset, such as crop yields, basic statistical methods, such as the PCA, may produce unreliable results. However, a robust version of the same method (rPCA) minimizes that common issue by recognizing abnormal observations first. In this study, we employ rPCA method in order to elucidate patterns inherent in the global year-to-year yield variability and find countries whose inter-annual yield variability tend to be more similar/opposite, as well as separating out the outlier yield estimates. The latter especially is a special outcome from the rPCA[5]. rPCA extracts orthogonal modes of year-to-year variability of crop yields (or concurrent variability of multiple national yields) from the high spatial and temporal variance in global yield dataset (in principal components/PCs) after skimming out the multi-variate outliers from the dataset and storing them in a sparse matrix.

In summary, rPCA decomposes a rectangular matrix M into a low-rank component matrix (L), and a sparse component matrix (S), by solving a convex optimization program called Principal Component Pursuit[70]. The schematic input and output data acquired from rPCA is presented in Figure S2. The S-matrix contains all the simultaneous yield outliers across all the countries in every year and L-matrix exhibits "new" interannual standardized yield anomalies for all the 85 countries. The approach to compute the sparse matrix (S) also minimizes the rank of the original data matrix (M). Therefore, the number of principal components would be a lesser number than an ordinary PCA applied on M-matrix and hence the total number of the PCs in our case is 26 (Figure S3).

SM4. Method to investigate leading modes of world-wide wheat yield variability: Following Figure S2, M is the standardized yield matrix (as in section SM3) where the low rank matrix (L) delivers standardized annual yield variability after removing yield outliers from M and storing in S. The L matrix have a number of vectors, specific to each country yield, on which the PCA method is run. We used the first four PC outputs to study characteristics of concurrent yields and their connections to climate variability while the error bars of the eigenvalues by Leave-1 out cross validation bootstraps for all the first 10 PCs are shown in Figure S5. It is important to note here that, fourth PC is not entirely separable from the others, and therefore cannot be interpreted physically apart from the following PCs.

SM5. Countries jointly contributing to each PC: Figure 2 indicated that countries with similar directional variability in annual yields (positively covarying nations that are also having high loading values of the same sign) are assigned with the same color while those co-varying but in a different manner (high loading values of opposite signs) are indicated by the opposite colors. The histograms of all the loading values (assigned to 85 nations) corresponding to the first 4 PCs are shown in Figure S6. The vertical blue and orange lines are based on statistical mean ± one standard deviation values of 85 loading values. Countries whose loading values are greater (absolute) than these standard deviation values are highlighted by the orange or blue colors indicating highly co-varying countries (Table S1, Figure 2), designated as PCon# (where # = PC number). It is important to note that wheat yields in each PCon# group synergistically vary but in variable proportions. PCon1 group included Nepal, Syria, DR Congo, Kenya, Niger, Tanzania, Tunisia, Austria, Bulgaria, Denmark, France, Germany, Greece, Hungary, Portugal, Romania, Sweden, Switzerland, New Caledonia, Bolivia, Ecuador, Paraguay and Venezuela. PCon2 group included Iran, Lebanon, Oman, Syria, Taiwan, Turkey, Yemen, Honduras, USA, Algeria, Niger, Rwanda, South Africa, Sudan, Tanzania, Cyprus, Denmark, Greece, Ireland, Italy, Netherlands, Portugal, Romania, Australia, Bolivia, Ecuador, and Colombia. PCon3 group included Afghanistan, Iran, Israel, Jordan, Myanmar, North Korea, Pakistan, Syria, Turkey, Canada,

Honduras, USA, Algeria, Malawi, Burundi, DR Congo, Kenya, Mauritania, Namibia, Niger, South Africa, Tunisia, Cyprus, Finland, Sweden, Albania, Chile, Uruguay, Ecuador, and Venezuela. PCon4 group included India, Japan, Myanmar, Oman, Yemen, Canada, Angola, Lesotho, Mauritania, Mozambique, Nigeria, Zambia, Bulgaria, Finland, France, Germany, Malta, Norway, Spain, Switzerland, Ecuador, and Venezuela (Table S1 provides more details).

SM6. Data for air temperature anomaly (ATa): The importance of air temperature on wheat yield has been discussed in numerous studies[6-11]. Here, we used monthly air temperature anomalies data (ATa) from 1963-2010 from University of Delaware Air Temperature database at 0.5-degree resolution. We downloaded the data from NOAA/OAR/ESRL PSD, Boulder, CO, USA, from their web site at https://www.esrl.noaa.gov/psd/ for the wheat cropland grid cells (Figure S12).

SM7. Data for Palmer Drought Severity Index (PDSI): PDSI was developed by Palmer[12] to measure the cumulative departure in surface water balance. Here, we evaluated correlations between local PDSI variability and the PCs. PDSI is highly correlated with soil moisture content[13], and an important factor influencing both rainfed and irrigated crop yields[14]. PDSI has a range between -10 (driest) to +10 (wettest) with values below -3 representing severe to extreme drought. The index has been widely used in studies assessing the impact of droughts on wheat [15-17]. We used gridded monthly self-calibrated PDSI data[18], at 2.5-degree resolution, from 1963-2010, obtained from NOAA/OAR/ESRL-PSD (http://www.esrl.noaa.gov/psd/), Boulder, CO, USA.

SM8. Data for Sea Surface Temperature anomalies (SSTa): SST is an essential parameter in weather prediction and atmospheric model simulations. We used monthly Extended Reconstructed Sea Surface Temperature (ERSST) version 4 dataset from 1963-2013, on global 2 x 2 grids downloaded from NOAA/OAR/ESRL PSD, Boulder, CO, USA, from their web site (https://www.esrl.noaa.gov/psd/).

SM9. Data for geopotential height (GPH): Geopotential height approximates heights of the pressure level in the upper atmosphere and its fluctuation drives the atmospheric circulation patterns. We have used GPH at 500 hPa pressure level (Z500), which is the most important variable describing the larger-scale air flows[19]. Detrended gridded monthly GPH data, at 2.5-degree resolution, was obtained from the same source as PDSI.

SM10. Data for larger-scale ocean-atmospheric indices: Ocean-atmospheric indices are used to characterize aspects of geophysical systems such as ocean-atmospheric circulation patterns that can trigger concurrent multiple regional climate variability across the globe (https://climatedataguide.ucar.edu/climate-data/overview-climate-indices)[20]. We obtained a range of monthly atmospheric and oceanic indices in time series format, downloaded from https://www.esrl.noaa.gov/psd/data/climateindices/list/. We categorized all these indices based on Pacific Ocean-based: Southern Oscillation Index (SOI), Extreme Eastern Tropical Pacific SST (Nino1+2), Eastern Tropical Pacific SST (Nino3), Central Tropical Pacific SST (Nino4), East Central Tropical Pacific SST (Nino3.4), Multivariate ENSO Index (MEI), Pacific/North American pattern (PNA), West Pacific (WP), Pacific Decadal Oscillation (PDO), Western Hemisphere Warm Pool (WHWP), Oceanic Nino Index (ONI); Atlantic-Pacific Ocean-based indices: Western Hemisphere Warm Pool (WHWP); and Atlantic Ocean-based indices: North Atlantic Oscillation (NAO), Tropical Southern Atlantic Index (TSA). The associations between some of these indices such as Nino, NAO and crops have been discussed in earlier studies[21-29].

SM11. Method to identify the major wheat producing-, exporting and importing-countries: We identified 20 major wheat producers, exporters and importers among 85 countries (Table S1) based on average annual wheat production, export and import quantities within the most recent decade (2001 - 2013). Like yields, we obtained all these data from FAOSTAT (http://www.fao.org/faostat/en/#data).

SM12. Method to define Most yield-Volatile Year (MVY) and Least yield-Volatile Year (LVY): A major assumption in standard statistical methods is that the data follows a known statistical distribution[30], but it is crucial to recognize the impacts of outliers on the outputs. Annual crop yield data recorded for the global countries can be influenced by many factors and among them climate is the chief, in general[31]. Subsequently, extreme climatic events, such as floods and droughts can reduce crop yields, which may not fall into a normal statistical distribution. Hence, it is likely that some unusual yield values (both low or high with respect to the mean within recent 7-year distribution) appearing across countries. We identified yield volatile years and countries contributing consistently to world-wide yield volatility such that we can investigate if such volatility is systematic having connections to climate (especially extremes).

There is rich body of literature relating to detection of outliers in various fields[4,5,30-32], background modeling from surveillance video is a well-known example. Despite having issues regarding outliers, as we mentioned above, to our knowledge, there hasn't been any effort to diagnose anomalous crop yield years. While outliers can be worrisome for some datasets, we realized that, sparse matrix (S-matrix) containing crop yield outliers, within multi-dimensional space, can provide relevant information to investigate global food supply volatility and losses because there may be a good reason for the co-incidence of extreme yield volatility, or not, within many countries. As such, we examined years when there were 10 largest and 10 smallest co-incidence of extreme yield departures (number of countries) from the global mean. To do that we used rPCA outputs stored in S-matrix (refer to SM3 & SM4), and defined the most yield-volatile years (MVY) as the years when there were top ten highest co-incidence of countries having significantly greater absolute yields in S-matrix (absolute national standardized yield larger than 1 standard deviation estimate of all the 85 national yields within the same year, Table S4). On the other hand, a least yield-volatile year (LVY) was characterized as the year when there were smallest number of nations having coinciding anomalous yields (Table S5). We eventually used these extreme 10 + 10 years from Tables S4 and S5 respectively to make composite maps of PDSI, ATa, and SSTa.

Here is a clear step-by-step process in detail to choose each MVY and LVY:

1. We consider sparse matrix (S in Figure S2)

2. For each year we compute standard deviation of all sparse values i.e. all the 85 countries participating (of course those having non-zero sparse yields).

3. Then we sort years based on the largest and the smallest standard deviation of yearly distributions in 2 where the widest/narrowest distributions defining MVY/LVY.

4. Then we remove countries whose absolute sparse value magnitudes in a year are less than the standard deviation of the corresponding year (Step 2) and hence the tails of yearly sparse yield distributions designate countries having extreme yield magnitudes in those years. We show the final results in the Tables S4 and S5.

5. Countries those repeatedly appeared in volatile years (step 3) are designated as most and least volatile countries, respectively, and are shown by thicker boundaries in the Figures 3 and 4.

SM13. Data on global wheat croplands coverage: In order to get the total coverage of wheat croplands we combined both irrigated (Figure S12-a) and rainfed grids (Figure S12-b)[33]. We used the combined map (Figure S12-c) to study local climate variability. We re-gridded every local climate data (PDSI and ATa) to match spatial scales of Figure S12-c.

SM14. Limitations to include wheat growing season in climate analysis: Wheat is not grown at fixed calendar dates and growing durations vary across the world[34]. Our analysis incorporated annual average climate variability, both at local as well as larger-scales, as opposed to locally-constrained "growing season", due to the following reasons:

First, the global yield dataset was complete and consistent for 85 countries from 1961-2013, on the annual time scale and national spatial scales. The same dataset did not separate out spring and winter wheat yields at the desired time span and/or spatial conditions. There was one higher (spatial) dimension yield dataset available, but for 1961-2008 with highest spatial dimension possible only for 17 countries in total, and so was not useful for our global assessment[35]. The data developers[35] also admitted that the frequency of the data reporting varied among countries and there were no separations in seasonal yields. Therefore, to confidently meet our research objectives, i.e. investigating co-variability in multi-national yields (and coinciding volatility), as many climates concurrently vary across the globe as a function of global climatic drivers, the best yield dataset we could possibly use was the FAO data at annual and national averages, which was complete and consistent for 85 nations over 1961-2013.

Second, we have used grids demonstrating combined irrigated and rainfed croplands across the globe (Figure S12) but did not use growing season dates specific for those grids. We obtained data on seasonal cropping calendars for both spring and winter wheat varieties from Sacks et al.[34] but we were unable to use that information for our climate analysis due to the below limitations:

1. SAGE dataset (http://www.sage.wisc.edu) provides mean growing season information for winter and spring wheat varieties (average planting and harvesting dates), focusing mainly on rainfed croplands[34] (Figure S13). Hence, this dataset missed out on many of the crucial croplands we have included in our study. Figure S12(c) indicates those included in our study (combined irrigated and rainfed croplands) across the globe while Figure S13 provides growing season information from the SAGE dataset. We particularly see that our cropland coverage in Figure S12 provides a much higher resolution and extent for local climate analysis, where a range of crucial wheat growing areas in south Asia (e.g. India, China) and eastern Europe are not covered in SAGE (Figure S13). We already faced earlier limitations due to yield data (un)availability within desired specifications and time frames, where Russia was excluded from our analysis due to Soviet Union breakdown issue. It was hence imperative not to lose more important producers from our analysis due to unavailability in growing season data. Inclusion of SAGE growing season dataset would only lead to exclusion of more countries in the eastern Europe and India from climate analysis, countries which were important for the PCs.

2. As evident from Figures S12 and S13, SAGE crop calendar observations generally applied to larger geographic regions, where, most observations were specified either for an entire country or for a fairly large sub-national units (e.g. at state-level within the USA). Countries, such as the USA, European region, Turkey, and China produces both winter and spring wheat (Figure S13), but yield data for these countries were mostly available as annual averages. In such a scenario,

deciding on which growing season to consider for local climate analysis was difficult. Furthermore, for larger-scale climate analysis to discover the common influence, we could only consider "one" consistent time scale. As a result, we ended up selecting "annual" time scale as the one and only that would be coherent across yield and climate variability. By incorporating both concurrent and previous year's climate variability we could possibly capture variable starting / harvesting dates (Figure S13) of local growing seasons to some extent. This, in our view, made sense, as Sacks et al.[50] indicated that wheat planting dates can be highly determined by a region's climate variability that is variable within and across countries.

3. SAGE does not capture any seasonal timing changes/shifts or variability within a given region. In reality, planting dates vary through space and time based on changes in the weather and climate (e.g. north-south gradient following temperature gradient), and also due to non-climatic factors such as soil properties, cultivar choice, farm management, changes in technological and socio-economic factors[34].

4. Finally, Sacks et al.[34] also mentioned that there may be some observations within SAGE dataset, where the growing season observations lacked an explicit label of "winter wheat" or "spring wheat". They also acknowledged that in tropical and subtropical regions this scheme could lead to mis-classification of spring vs winter cereals, which could be solved if a minimum temperature threshold was included to distinguish between either type, as winter variety requires cold temperatures for vernalization; however, the data developers[34] were not able to identify a robust threshold due to the reason that vernalization requirements differ between cultivars. As a result, Sacks et al.[34] made a strong cautionary note to the data users that *"..our data set should not be used to determine which regions actually grow winter versus spring cereals*.

References

1. Troy, T., Kipgen, C. & Pal, I. The impact of climate extremes and irrigation on us crop yields. *Environ. Res. Lett.* **10**, 054013 (2015).

2. Candes, E. J., Li, X., Ma, Y. & Wright, J. Robust principal component analysis? *J. ACM.* **58**,11:1–11:37 (2011).

3. Baeriswyl, P. A. & Rebetez, M. Regionalization of precipitation in Switzerland by means of principal component analysis. *Theoretical and Applied Climatology.* **58**, 31–41 (1997).

4. Jackson, D. A. & Chen, Y. Robust principal component analysis and outlier detection with ecological data. *Environmetrics.* **15**, 129–139 (2004).

5. Hubert, M., Rousseeuw, P. J. & Branden, K. V. Robpca: A new approach to robust principal component analysis. *Technometrics.* **47**, 64–79 (2005).

6. Lobell, D. B. & Field, C. B. Global scale climate-crop yield relationships and the impacts of recent warming. *Environ. Res. Lett.* **2**, 014002 (2007).

7. Reidsma, P., Ewert, F., Lansink, A. O. & Leemans, R. Adaptation to climate change and climate variability in European agriculture: The importance of farm level responses. *European Journal of Agronomy.* **32**, 91 – 102 (2010).

8. Asseng, S. *et al.* Uncertainty in simulating wheat yields under climate change. *Nature Climate Change.* **3**, 827 EP – (2013).

9. Deryng, D., Conway, D., Ramankutty, N., Price, J. & Warren, R. Global crop yield response to extreme heat stress under multiple climate change futures. *Environ. Res. Lett.* **9**, 034011 (2014).

10. Asseng, S., Foster, I. & Turner, N. C. The impact of temperature variability on wheat yields. *Global Change Biology.* **17**, 997–1012 (2011).

11. Gaudet, D. A., Laroche, A. & Yoshida, M. Low temperature-wheat-fungal interactions: A carbohydrate connection. *Physiologia Plantarum.* **106**, 437–444 (1999).

12. Palmer, W.: 1965, *Meteorological Drought*, U.S. Weather Bureau Research Paper. **45**, 58. (1965).

13. Dai, A., Trenberth, K. E. & Qian, T. A global dataset of palmer drought severity index for 18702002: Relationship with soil moisture and effects of surface warming. *Journal of Hydrometeorology.* **5**, 1117–1130 (2004).

14. Holzman, M., Rivas, R. & Piccolo, M. Estimating soil moisture and the relationship with crop yield using surface temperature and vegetation index. *International Journal of Applied Earth Observation and Geoinformation.* **28**, 181 – 192 (2014).

15. Li, Y., Ye, W., Wang, M. & Yan, X. Climate change and drought: a risk assessment of crop yield impacts. *Climate research.* **39**, 31–46 (2009).

16. Attavanich, W. & McCarl, B. The effect of climate change, CO_2 fertilization, and crop production technology on crop yields and its economic implications on market outcomes and welfare distribution. 2011 Annual Meeting, July 24-26, 2011, Pittsburgh, Pennsylvania 103324, Agricultural and Applied Economics Association (2011).

17. Narasimhan, B. & Srinivasan, R. Development and evaluation of Soil Moisture Deficit Index (SMDI) and Evapotranspiration Deficit Index (ETDI) for agricultural drought monitoring *Agricultural and Forest Meteorology.* **133**, 69 – 88 (2005).

18. Wells, N., Goddard, S. & Hayes, M. J. A self-calibrating palmer drought severity index. *Journal of Climate.* **17**, 2335–2351 (2004).

19. Weare, B. C. Use of long-range weather forecasts in crop predictions. *California Agriculture.* **44**, 28–29 (1990).

20. Steptoe, H., Jones, S. E. O. & Fox, H. Correlations between extreme atmospheric hazards and global teleconnections: Implications for multihazard resilience. *Reviews of Geophysics.* ***56**, 50–78* (2017).

21. Heino, M. et al. Two-thirds of global cropland area impacted by climate oscillations. *Nat. Commun*. **9**, 1257 (2018).

22. Iizumi, T. et al. Impacts of El Niño Southern Oscillation on the global yields of major crops. *Nat. Commun.* **5**, 3712 (2014).

23. Gonsamo, A. & Chen, J. M. Winter teleconnections can predict the ensuing summer European crop productivity. *Proc. Natl Acad. Sci.* **112**, E2265–E2266 (2015).

24. Yuan, C. & Yamagata, T. Impacts of IOD, ENSO and ENSO Modoki on the Australian winter wheat yields in recent decades. *Sci Rep.* **5**, 17252–17252 (2015).

25. Nguyen-Huy, T., Deo, R. C., Mushtaq, S., An-Vo, D.-A. & Khan, S. Modeling the joint influence of multiple synoptic-scale, climate mode indices on Australian wheat yield using a vine copula-based approach. *European Journal of Agronomy.* **98**, 65 – 81 (2018).

26. Lu, W., Atkinson, D. E. & Newlands, N. K. ENSO climate risk: predicting crop yield variability and coherence using cluster-based PCA. *Modeling Earth Systems and Environment.* **3**, 1343– 1359 (2017).

27. Persson, T., Bergjord, A.K. & Höglind, M. Simulating the effect *of the* North Atlantic Oscillation on frost injury in winter wheat. *Climate Research.* **53**, 43–53 (2012).

28. Cantelaube, P., Terres, J.M. & Doblas-Reyes, F. J. Influence of climate variability on European agriculture-Analysis of winter wheat production. *Climate Research.* **27**, 135–144 (2004).

29. Kettlewell, P. S., Stephenson, D. B., Atkinson, M. D. & Hollins, P. D. Summer rainfall and wheat grain quality: Relationships with the North Atlantic Oscillation. *Weather.* **58**, 155–164 (2003).

30. Najafi, E., Devineni, N., Khanbilvardi, R. M. & Felix, K. Understanding the changes in global crop yields through changes in climate and technology. *Earth's Future.* **6**, 410–427 (2018).

31. Osborne, T. M. & Wheeler, T. R. Evidence for a climate signal in trends of global crop yield variability over the past 50 years. *Environ. Res. Lett.* **8**, 024001 (2013).

32. Frugone-lvarez, M. *et al.* A 7000-year high-resolution lake sediment record from coastal central Chile (Lago Vichuquén, 34° S): implications for past sea level and environmental variability *Journal of Quaternary Science.* **32**, 830–844 (2017).

33. ORNL DAAC. Spatial data access tool (SDAT) (2017).

34. Sacks, W. J., Deryng, D., Foley, J. A. & Ramankutty, N. Crop planting dates: an analysis of global patterns. *Global Ecology and Biogeography.* **19**, 607–620 (2010).

35. Ray, D. K., Gerber, J. S., MacDonald, G. K. & West, P. C. Climate variation explains a third of global crop yield variability. *Nat. Commun*. **6**, 5989 (2015).

Supplementary Figures' Captions

Fig. S1. Two histograms showing statistical distributions of standardized wheat yields recorded for 85 producing nations in 1998 (red) and 2007 (blue) respectively. Purple indicates the overlap. 1998 (2007) had a positively (negatively) skewed global yield distribution and globally surplus (deficit) yields/supply. 1998 had relatively lower (higher) volatility (measured by global standard deviation estimate marked in red (and blue) color lines. 2007 was a record yield-volatile year with a greater negative skew and major supply deficits.

Fig. S2. Original (M), low rank (L) and sparse (S) matrix of standardized wheat yields for 85 countries (on x-axis) from 1964 to 2010 (on y-axis), and z-axis indicating standardized yields (unit-less).

Fig. S3. The cumulative variance explained (in %) by the leading modes of global yield variability or principal components (PC).

Fig. S4. The concurrent and one-year-lagged Spearman correlations between PC5-10 and climate variability. As Figure 2, PDSI and ATa indicate local climate while SSTa indicates larger-scale climate driver. The locations with statistically significant correlations at the 95% levels are designated as small black dots over the wheat cropland areas and the same over the global oceans are indicated by appropriate colors. The orange and blue colored country boundaries indicate countries with high loading values specific to a PC, indicating highly co-varying nations.

Fig. S5. The error bars of the eigenvalues by leave-1 out cross validation bootstrap for the first 10 PCs.

Fig. S6. The histograms of loading values corresponding to first 4 principal components and 85 nations and fitted normal density functions. The vertical blue and orange lines are based on closer to ± one standard deviation. In Figure 2, countries whose loading values are greater (or smaller) than this one standard deviation values are highlighted by the orange or blue color boundaries, indicating highly co-varying countries in yields (names are indicated in Table S1).

Fig. S7. The spearman rank correlation patterns between (a) PC1, (b) PC2, (c) PC3, and (d) PC4 and standardized national yields in low rank matrix (L = M – S in Fig S2). Countries that are not within the scope of this study are highlighted by gray color.

Fig. S8. Country-specific growing area (in percentage) influenced by the local climate variability (concurrent-year ATa, lagged-ATa, concurrent-year PDSI, and lagged-PDSI variability), as exhibited by significant Spearman rank correlations at 95% level between local climate indicators and leading PCs (as displayed in Figure 2). Only PCon# are marked here.

Fig. S9. The cropland area (on logarithmic scale) influenced by ATa and PDSI and their combination in hPcon#.

Fig. S10. Histograms of the sparse values (S-matrix) from 1964 to 2010 (without zero values). The most yield-volatile years (MVY) have negative sparse values and are marked in red while the opposite is found for the least yield-volatile years (LVY).

Fig. S11. Boxplots of the yearly sparse values (S-matrix) from 1964 to 2010 (excluding zeroes). The most and least yield volatile years are marked in yellow and green colors respectively.

Fig. S12. Global coverage of (a) irrigated, (b) rainfed and (c) combination of both irrigated and rainfed wheat croplands.

Fig. S13: Growing season information for spring and winter wheat varieties (a) planting start dates for spring, (b) planting end dates for spring, (c) planting start dates for winter, (d) planting end dates for winter, (e) harvesting start dates for spring, (f) harvesting end dates for spring, (g) harvesting start dates for winter, (h) harvesting end dates for winter, (i) combination of all of them showing spatial coverage[51].

Supplementary Tables

Table S1. Countries corresponding to each PC (orange and blue highlights in Figure 2), and their ranks as producer, exporter or importers (e.g. 17e for Austria indicates country rank 17 as an exporter, method to choose country ranks is discussed in section SM11). Countries showing significant local climate influence in Figure 2 (correlations between PCs and local climate indicators) is denoted here by a star within the orange & blue highlights.

Country	major exporter/importer/ producer rank based on the average production from 2001 to 2013			PC1	PC2	PC3	PC4	Country	major exporter/importer/ producer rank based on the average production from 2001 to 2013			PC1	PC2	PC3	PC4
Austria	17e			*				Argentina	5e		12p				
Bulgaria	10e			*				Bolivia							
Cyprus								Brazil	12e	3i	18p				
Denmark	13e		19p	*	*			Chile							
Finland								Colombia							
France	2e		4p	*			*	Ecuador							
Germany	6e	14i	6p	*			*	Paraguay	19e			*			
Greece								Peru		20i					
Hungary	9e							Venezuela							
Ireland								Uruguay	20e						
Italy		2i	15p					Australia	4e		8p		*		
Malta								New Caledonia							
Netherlands		7i						New Zealand							
Poland	14e		13p					Iran		13i	11p		*	*	
Portugal								Iraq		15i					
Romania	11e		17p	*				Israel							
Spain	18e	6i	16p				*	Jordan							
Sweden								Lebanon							
UK	7e		10p					Oman							
Albania								Saudi Arabia							
Norway								Syria							
Switzerland								Yemen		17i			*		*
Turkey		18i	9p		*	*		Afghanistan							
Algeria		4i				*		Bangladesh		16i					
Angola								Bhutan							
Burundi								India	8e		2p				*
DR Congo								Nepal							
Egypt		1i	14p					Pakistan			7p			*	
Kenya								Myanmar							
Lesotho								China	16e	11i	1p				
Libya								Taiwan							
Malawi								North Korea							
Mauritania								Japan		5i					*
Morocco		12i	20p					Mongolia							
Mozambique								South Korea		8i					
Namibia															
Niger		10i				*									
Nigeria															
Rwanda															
Sudan															
South Africa															
Tunisia															
Tanzania															
Zambia															
Zimbabwe															
Canada	3e		5p			*	*								
Mexico	15e	9i													
USA	1e	19i	3p		*	*									
Guatemala															
Honduras															

Table S2. Spearman rank correlation coefficients between each PC time series and standardized national yields in L matrix (also shown on a map in Figure S7). Correlations (r), once squared (r^2), indicate to the extent by which each PC explains national yield variance or vice versa. For example: the global variability captured within PC1 (10.5%) associates highly with Austria but the same with the other 3 PCs is minimal. On the other hand, PC1 illustrates a higher correlation with Bulgaria's yields (0.74) while PC4 indicating an opposite type of influence.

Country	PC1	PC2	PC3	PC4	Country	PC1	PC2	PC3	PC4
Austria	0.71	0.16	0.11	0.21	Argentina	0.16	-0.29	0.17	-0.05
Bulgaria	0.74	-0.28	-0.06	-0.36	Bolivia	-0.31	0.34	0.17	0.21
Cyprus	-0.16	-0.33	0.58	0.19	Brazil	-0.15	-0.24	-0.26	0.26
Denmark	0.71	0.33	0.15	-0.14	Chile	-0.15	0.14	0.36	-0.05
Finland	0.38	0.12	0.51	-0.47	Colombia	0.07	0.56	0.30	0.25
France	0.65	0.22	0.19	0.51	Ecuador	-0.27	-0.42	0.43	0.26
Germany	0.68	0.30	0.16	0.35	Paraguay	-0.35	-0.19	-0.19	0.08
Greece	0.50	-0.40	0.01	-0.20	Peru	0.39	0.01	0.28	-0.12
Hungary	0.78	0.02	0.11	0.12	Venezuela	-0.36	0.10	0.48	0.34
Ireland	0.52	0.37	-0.20	-0.21	Uruguay	0.07	0.37	-0.37	0.03
Italy	0.21	-0.38	-0.18	0.19	Australia	-0.11	0.76	0.08	0.04
Malta	0.49	-0.14	0.00	-0.41	New Caledonia	-0.62	-0.24	-0.12	0.28
Netherlands	0.46	0.39	0.17	0.14	New Zealand	0.20	0.05	0.09	0.11
Poland	0.57	0.37	0.09	0.22	Iran	-0.11	-0.43	-0.33	0.03
Portugal	0.41	-0.51	0.11	0.19	Iraq	0.30	-0.12	0.23	-0.10
Romania	0.86	-0.22	0.09	-0.17	Israel	-0.10	0.04	0.66	0.00
Spain	0.39	-0.26	-0.05	0.54	Jordan	0.02	0.06	0.40	0.15
Sweden	0.57	0.28	0.51	-0.02	Lebanon	0.18	-0.49	0.29	-0.21
UK	0.55	0.36	0.01	0.06	Oman	0.15	0.39	0.07	-0.49
Albania	0.55	0.14	-0.15	0.06	Saudi Arabia	-0.21	0.21	-0.16	-0.07
Norway	0.41	0.30	0.15	0.47	Syria	-0.20	-0.58	0.60	-0.02
Switzerland	0.67	0.10	0.13	0.49	Yemen	-0.01	-0.60	0.19	-0.38
Turkey	0.45	-0.54	-0.31	-0.24	Afghanistan	0.29	-0.05	-0.41	0.17
Algeria	-0.18	-0.49	0.27	-0.18	Bangladesh	-0.03	0.27	0.18	-0.14
Angola	0.08	-0.29	-0.18	0.50	Bhutan	0.33	-0.02	-0.25	-0.34
Burundi	-0.31	-0.15	-0.30	0.27	India	0.41	0.26	0.34	-0.30
DR Congo	-0.38	-0.02	0.31	-0.17	Nepal	-0.65	0.00	0.06	-0.31
Egypt	0.16	0.26	-0.07	-0.14	Pakistan	-0.14	-0.24	-0.71	0.16
Kenya	-0.33	0.19	-0.28	-0.13	Myanmar	0.17	-0.29	0.74	-0.40
Lesotho	0.12	-0.42	0.01	-0.44	China	0.27	0.02	0.14	0.00
Libya	0.63	-0.15	-0.07	-0.22	Taiwan	-0.13	-0.43	0.30	0.09
Malawi	0.17	-0.05	-0.68	0.13	North Korea	-0.08	0.33	-0.57	0.21
Mauritania	0.28	0.04	0.48	-0.44	Japan	0.08	0.08	0.08	0.64
Morocco	0.20	-0.27	0.12	0.03	Mongolia	0.49	0.29	-0.23	-0.24
Mozambique	-0.04	0.18	0.14	-0.50	South Korea	-0.25	0.14	-0.01	0.19
Namibia	0.38	0.05	-0.46	0.12					
Niger	-0.29	-0.58	-0.18	0.20					
Nigeria	0.33	-0.32	0.30	-0.72					
Rwanda	-0.19	0.34	0.00	0.21					
Sudan	-0.09	-0.60	-0.13	0.33					
South Africa	-0.20	0.34	0.31	0.22					
Tunisia	-0.44	-0.32	0.60	0.07					
Tanzania	-0.31	0.31	-0.10	-0.01					
Zambia	0.31	-0.16	0.35	-0.44					
Zimbabwe	0.30	0.09	-0.23	-0.15					
Canada	0.25	0.15	-0.31	-0.51					
Mexico	-0.07	-0.02	-0.12	0.07					
USA	-0.05	0.42	-0.30	0.02					
Guatemala	0.02	0.07	0.36	-0.10					
Honduras	0.10	-0.33	0.43	0.18					

Table S3. Spearman rank correlations between each PC time series and larger-scale ocean-atmospheric indices. Correlations indicate magnitudes of explanation of PCs by each pattern. For example: The Pacific indices indicate greatest degree of explanation of the variance of PC1, while western Pacific on PC3, and tropical southern Atlantic index on PC4. The significant correlations based on 90% and 95% confidence intervals are highlighted by light and dark brown colors respectively.

Location	Monthly Atmospheric and Ocean Time Series		The months that are averaged	correlation of the index with PC1	correlation of the index with PC2	correlation of the index with PC3	correlation of the index with PC4
Pacific Ocean	PNA	Pacific North American Index	Annual	-0.366			
			DJF				
	WP	Western Pacific Index	Annual			-0.381	
			DJF				
	SOI	Southern Oscillation Index	Annual				
			DJF	0.475	-0.298		
	BEST	Bivariate ENSO Timeseries	Annual				
			DJF	-0.480	0.247		
	WHWP	Western Hemisphere warm pool	Annual	-0.253			
			DJF				
	PDO	Pacific Decadal Oscillation	Annual	-0.283			
			DJF	-0.307			
	ONI	Oceanic Nino Index	Annual				
			DJF	-0.453			
	MEI	Multivariate ENSO Index	Annual				
			DJF	-0.441	0.244		
	TPI (IPO)	Tripole Index for the Interdecadal Pacific Oscillation	Annual				
			DJF	-0.426			
	TNI	Trans-Niño Index	Annual				
			DJF	0.312			
	Globally Integrated	Globally Integrated Angular Momentum	Annual				
			DJF	-0.360	0.280		
	Nino 3	Eastern Tropical Pacific SST	Annual				-0.251
			DJF	-0.405	0.290		
	Nino 3.4	East Central Tropical Pacific SST	Annual				
			DJF	-0.459			
	Nino 4	Central Tropical Pacific SST	Annual				
			DJF	-0.452			
	Nino 1+2	Extreme Eastern Tropical Pacific SST	Annual				-0.247
			DJF	-0.256	0.285		
	EP NP	East Pacific/North Pacific Oscillation	Annual	-0.323			
			JFM				
Atlantic Ocean	NAO	North Atlantic Oscillation	Annual	0.246			
			DJF				
	AO	Atlantic Oscillation	Annual	0.251			
			DJF	0.268		0.251	
	SCP	Scandinavian Pattern	Annual				
			DJF		-0.267		
	EAP	East Asia/Pacific	Annual	-0.288		-0.279	
			DJF				
	TSA	Tropical Southern Atlantic Index	Annual				0.315
			DJF		0.287		0.391
	CAR	Caribbean SST Index	Annual	-0.245			
			DJF				

Table S4. World-wide top 10 most yield volatile years within 1964-2010 and countries contributing to global volatility. Years when 10 largest co-incidence of extreme yield departures (countries) happened from the global mean indicating most yield-volatile years (MVY) (absolute national yields greater than 1 standard deviation estimate of all the 85 national yields within the same year). These extreme 10 years are later used to make composite maps of PDSI, ATa, SSTa, and GPH – Figures 3 and 4.

**Most volatile years**	**1977**	**2007**	**2000**	**1964**	**1991**	**1968**	**1999**	**1979**	**1993**	**2002**
**Number of countries with surplus**	8	7	5	11	4	10	6	5	10	8
**Number of countries with deficit**	14	13	10	9	9	8	11	18	9	13
Standrad deviation	0.84	0.79	0.76	0.75	0.75	0.74	0.72	0.71	0.71	0.71
New Zealand	0.90	0.97					-0.76	-1.05	0.86	
Paraguay		-1.96			-1.40	-1.71		1.07	0.82	
North Korea	1.04		-1.81	0.78		-0.93				
Austria			-1.09				1.20	-1.28	-1.79	
Cyprus	-1.37		-1.47		-1.38					0.98
Greece	-1.52		0.98	0.76		-1.12				
Jordan				1.31			1.80	-1.00	1.06	
Turkey		-0.89					-1.08	0.95	0.93	
Morocco	-1.68	-1.30	-1.08			1.00				
Australia	-0.99	-0.84				0.86				-1.40
Uruguay	-1.31			1.50		-1.72				-1.55
Argentina		-0.89			0.86	-0.86			1.15	
Nepal		1.23	1.49			0.82				-0.85
Myanmar			1.01	-0.88		1.16			-0.83	
Mozambique			-1.48		0.87				-0.87	0.91
Nigeria	0.90			-1.43					0.94	-0.84
Tanzania	-1.29				-1.33				-1.05	-0.91
Japan					-1.66	1.15		1.14		
South Korea	-0.84		-1.44						-0.89	
Malta						-1.46	-1.13			1.67
Poland			-0.97	-1.31				-0.93		
Sweden		1.18		0.81						0.77
UK		-1.24				-1.55				0.74
Iran			-0.81	-1.05				1.13		
Iraq						0.92		-0.80		1.11
Israel				0.76			-1.40	-0.98		
Mexico	-1.01			1.48						1.14
Albania					-0.90		-0.91		0.98	
Switzerland	-1.37						-1.25	0.89		
Colombia				-1.70		0.76				-0.77
Kenya	0.90		-0.77		-1.44					
Honduras					-1.79					-1.26
Mongolia							-1.18	-0.97		
Denmark						1.13				-0.72
Finland		1.30					-1.73			
Ireland						1.57			-1.49	
Italy	-1.55	-1.31								
Netherlands		-1.33								-0.72
Lebanon		-1.70						-1.10		
Yemen					-1.61		1.08			
Egypt				1.07				-0.93		
Tunisia					1.16			-1.07		
Canada		-1.01						-0.86		
New Caledonia			1.21					-1.49		
Norway									1.15	-1.10

Ecuador		0.99		-1.03						
Peru					-1.94		0.85			
Bhutan			0.89	-1.75						
India	-1.13								-1.21	
Pakistan				0.76				-1.66		
Angola	1.25						-1.05			
DER Congo		0.97							-1.42	
Lesotho	-1.45				0.78					
Libya		2.00								-1.79
Malawi	1.54							-1.07		
Mauritania			-1.03	-0.88						
Rwanda							-1.45	-1.65		
South Africa	1.63									0.80
Zimbabwe				1.00					0.98	
Guatemala									-0.79	
Taiwan							1.19			
Bulgaria		-0.90								
France		-1.19								
Germany		-0.90								
Hungary								-1.14		
Romania								-0.80		
Spain							-1.67			
Oman				-0.78						
Syria									0.72	
Algeria						1.10				
USA										-1.80
Brazil	0.95									
Chile	-1.26									
Venezuela	-1.23									
Burundi								-1.16		
Namibia						-1.26				
Niger										-0.88
Sudan				1.34						
Zambia							0.90			
China										
Portugal										
Saudi Arabia										
Bolivia										
Afghanistan										
Bangladesh										

Table S5. World-wide least yield volatile years within 1964-2010 and countries contributing to their volatility. Years when 10 smallest co-incidence of extreme yield departures (countries) happened from the global mean indicating least yield-volatile years (MVY) (absolute national yields greater than 1 standard deviation estimate of all the 85 national yields within the same year). These least extreme 10 years are also used to make composite maps of PDSI, ATa, SSTa, and GPH – Figures 3 and 4.

**Least volatile years**	**1986**	**1975**	**2004**	**1982**	**1981**	**1965**	**1969**	**1988**	**1967**	**1992**
**Number of countries with surplus**	19	10	12	14	8	10	9	11	12	8
**Number of countries with deficit**	3	5	5	3	6	7	7	9	9	9
Standrad deviation	0.44	0.49	0.50	0.54	0.57	0.57	0.58	0.58	0.58	0.60
Sudan			-0.89	0.79	1.35	-0.73		0.72	0.71	
Mongolia	0.54	1.40					-1.04	0.58	1.11	
Austria	-0.61		0.82			-1.06		0.86		1.59
Cyprus		0.50	-0.90			0.83		0.63	0.70	
Morocco	0.49			0.75	-1.83			0.65	-0.59	
Mozambique			-1.08	0.66		0.67	0.69	-0.67		
Zambia	1.16				-0.83	0.73	1.63			-0.89
Egypt		0.63	1.16				-0.83		-0.66	
Canada	0.50			0.75				-0.90	-0.98	
Australia				-1.33		-0.72		0.74	-0.61	
Colombia			0.58	1.57	0.66					-0.64
Pakistan			0.58			0.74	-1.23		-0.85	
South Africa	0.75			-1.04	0.98			-1.06		
Bulgaria		-0.76	0.77	0.62						
Ireland	-0.66		1.63				0.83			
Netherlands	1.09			0.69					0.91	
Poland			0.83					-0.75		-0.66
Syria	0.58				1.14			0.71		
Yemen		0.78			0.58				0.65	
Turkey				1.09		-0.91		1.16		
Tunisia		0.50		0.93						0.75
Algeria		1.07						-0.70		0.63
Mexico			-0.55	0.85				-0.59		
Uruguay				0.79	-0.86					1.47
Bolivia		-0.68		-0.82					-0.59	
Brazil			0.54		-0.76		-0.68			
Lesotho			-0.81			0.94				0.69
Niger				1.35	0.61				1.45	
Tanzania						0.68		0.62	-0.65	
Guatemala		-0.81							-1.08	
North Korea	0.44					-0.64				
Japan	0.50				-0.97					
South Korea							0.83	0.91		
Portugal		0.53						-0.88		
Spain		0.83						0.92		
Iran	0.54				-1.43					
Israel			0.65						0.94	
Jordan						0.64	1.15			
Lebanon			0.53			-1.38				
Saudi Arabia		0.66				0.58				
New Caledonia				0.84					0.99	
New Zealand		-1.11							0.78	
Norway								-1.29		-0.91
Chile							1.23			-0.66
Ecuador		-1.26							0.73	

Paraguay			1.00				0.58			
Peru						1.32	-0.63			
Venezuela							0.76		0.81	
Angola	0.57						-0.79			
Burundi	0.54						0.67			
Mauritania					0.94					-0.64
Rwanda								-1.42		1.50
Zimbabwe	1.04									-0.75
Honduras							-1.03			
Taiwan					0.78					
China	0.47									
Denmark										-0.88
Finland										-1.44
Germany	0.48									
Italy		0.60								
Malta				0.80						
UK	0.48									
Iraq									-1.24	
Oman						-0.98				
USA										0.66
Albania	-0.68									
Argentina						1.75				
Bhutan			0.69							
India									0.78	
Nepal	1.06									
Myanmar	0.58									
Libya	0.79									
Malawi										1.20
France										
Greece										
Hungary										
Romania										
Sweden										
Switzerland										
Afghanistan										
Bangladesh										
DR Congo										
Kenya										
Namibia										
Nigeria										

Table S6. A list with hPCon# countries in which a 1-year lagged local climate indicator is relevant (Figure 2) due to crop calendar (Figure S13). Crop calendar data is bi-weekly, from source: https://asheshwor.shinyapps.io/cropcal/ based on SAGE data[50] on country basis (https://nelson.wisc.edu/sage/data-and-models/crop-calendar-dataset/index.php).

Country	Type of wheat	State/Province	PC1	PC2	PC3	PC4	Crop Calender											
							Jan	Feb	Mar	Apr	May	Jun	Jul	Aug	Sep	Oct	Nov	Dec
Australia		New South Wales		X														
		Queensland																
		South Australia																
		Victoria																
		Western Australia																
India	Wheat (Rabi)	Gujarat				X												
		Gujarat																
		Himachal Pradesh																
		Karnataka																
		Maharashtra																
		Rajasthan																
		West Bangal																
Pakistan	Winter wheat				X													
Japan						X												
Iran				X	X													
Austria		No data	X															
Bulgaria	Winter wheat		X															
Romania			X															
Spain	Durum wheat					X												
	Soft wheat																	
Denmark	Winter wheat		X	X														
France	Winter wheat		X			X												
Germany	Winter wheat		X			X												
Turkey				X	X													
	Spring wheat																	
Yemen		No data		X		X												
Algeria					X													
Niger		No data			X													
Canada		No data			X	X												
USA	Winter wheat	North Dakota		X	X													
	Spring wheat	North Dakota																
	Winter wheat	Kansas (Top state producer)																
	Winter wheat	Florida																
Paraguay			X															

Plant	
Growth	
Harvest	

Figure S1

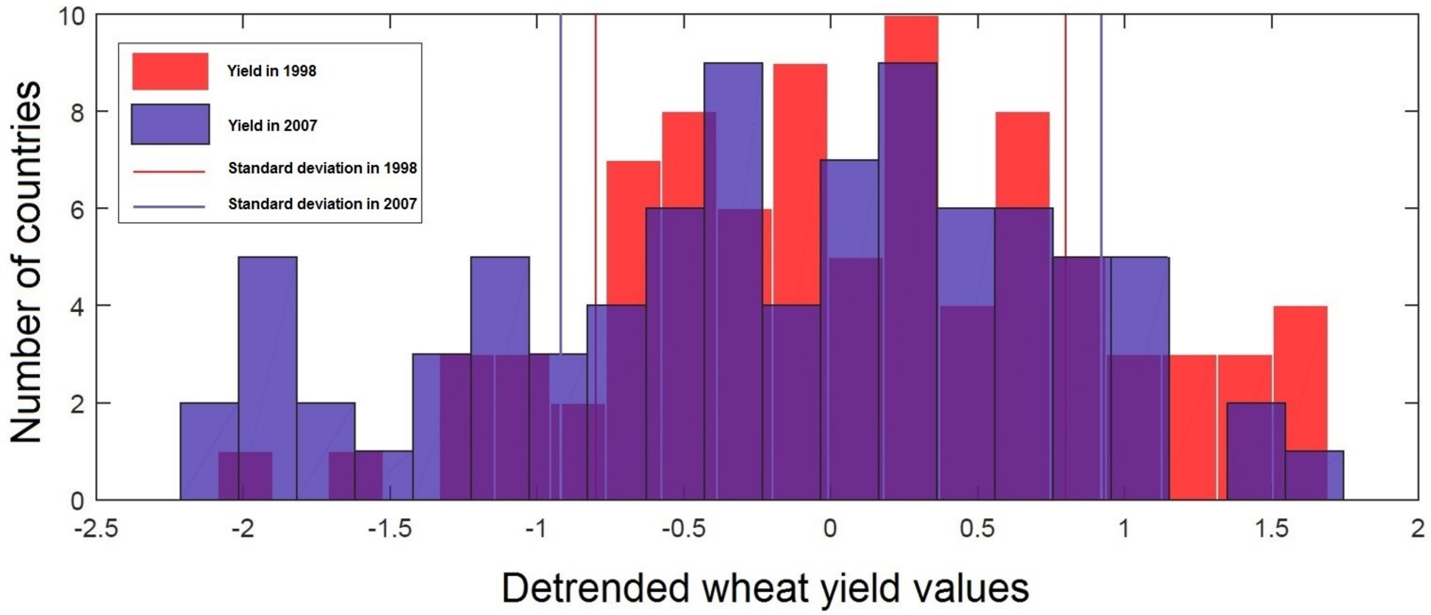
Yield in 1998
Yield in 2007
Standard deviation in 1998
Standard deviation in 2007
Number of countries
Detrended wheat yield values
0
2
4
6
8
10
-2.5
-2
-1.5
-1
-0.5
0
0.5
1
1.5
2

Figure S2

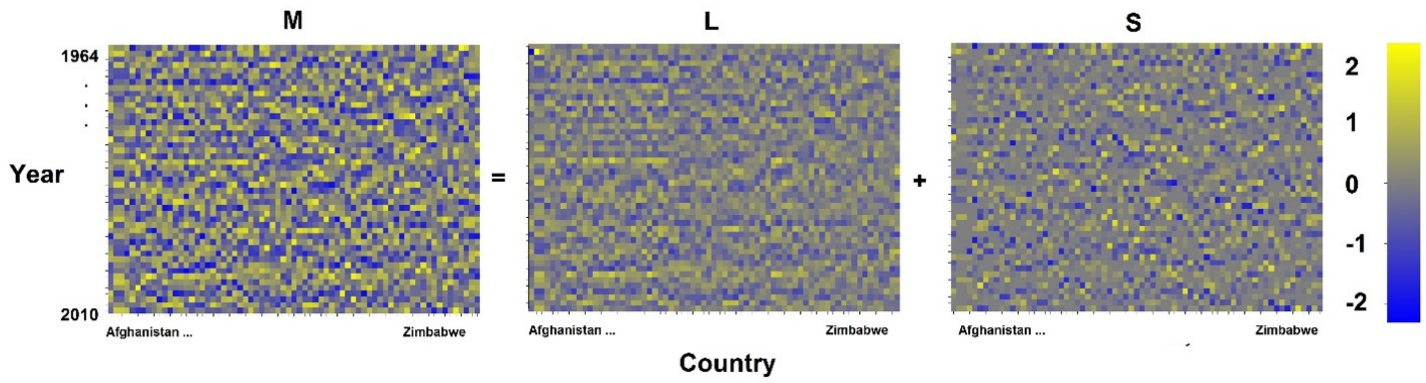
M
L
S
1964
.
.
.
Year
2010
=
+
Afghanistan ...
Zimbabwe
Afghanistan ...
Zimbabwe
Afghanistan ...
Zimbabwe
Country
2
1
0
-1
-2

Figure S3

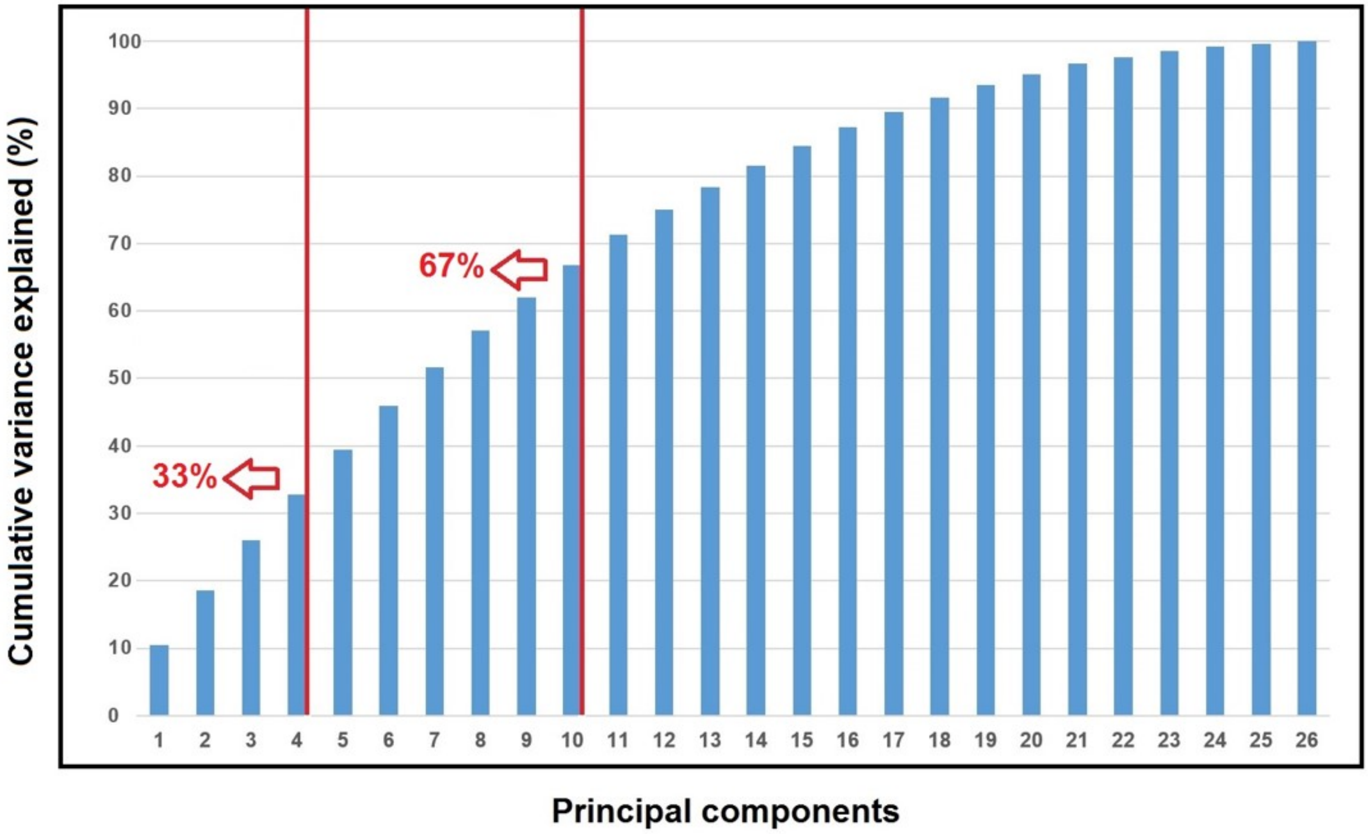

Cumulative variance explained (%)
100
90
80
70
60
50
40
30
20
10
0
67%
33%
1
2
3
4
5
6
7
8
9
10
11
12
13
14
15
16
17
18
19
20
21
22
23
24
25
26
Principal components

Figure S4

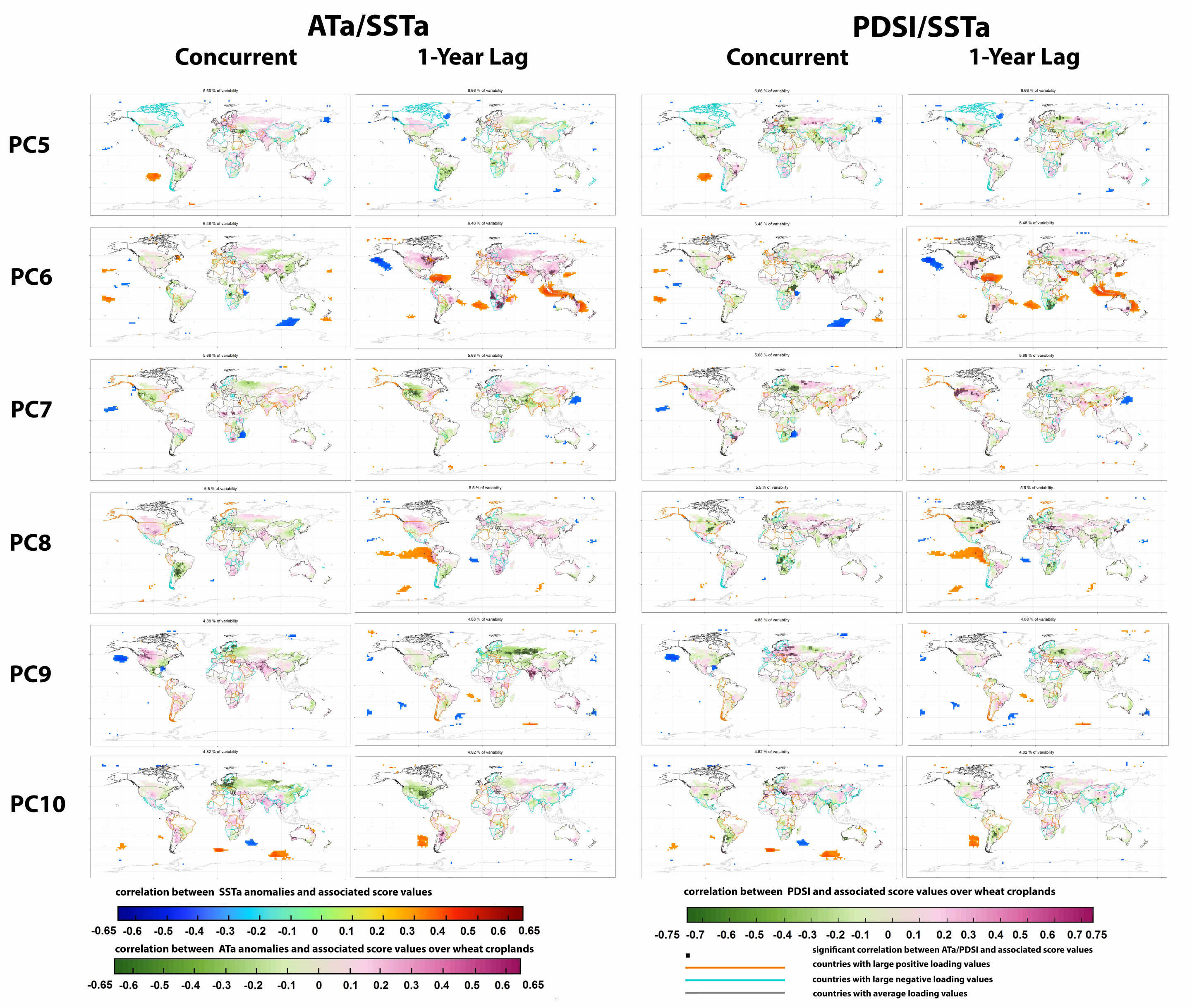

ATa/SSTa
Concurrent
1-Year Lag
PDSI/SSTa
Concurrent
1-Year Lag
PC5
PC6
PC7
PC8
PC9
PC10
correlation between SSTa anomalies and associated score values
-0.65 -0.6 -0.5 -0.4 -0.3 -0.2 -0.1 0 0.1 0.2 0.3 0.4 0.5 0.6 0.65
correlation between ATa anomalies and associated score values over wheat croplands
-0.65 -0.6 -0.5 -0.4 -0.3 -0.2 -0.1 0 0.1 0.2 0.3 0.4 0.5 0.6 0.65
correlation between PDSI and associated score values over wheat croplands
-0.75 -0.7 -0.6 -0.5 -0.4 -0.3 -0.2 -0.1 0 0.1 0.2 0.3 0.4 0.5 0.6 0.7 0.75
significant correlation between ATa/PDSI and associated score values
countries with large positive loading values
countries with large negative loading values
countries with average loading values

Figure S5

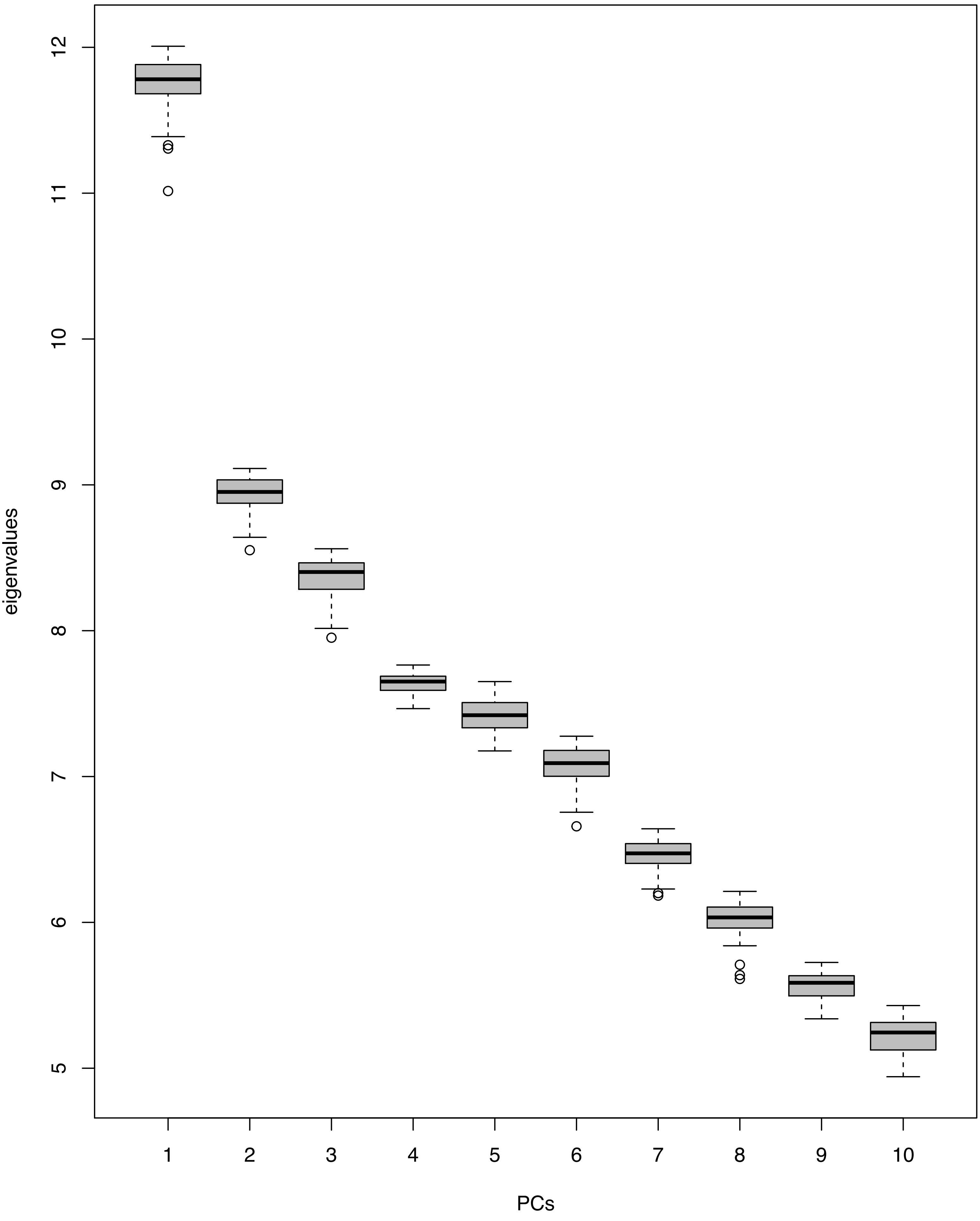

Figure S6

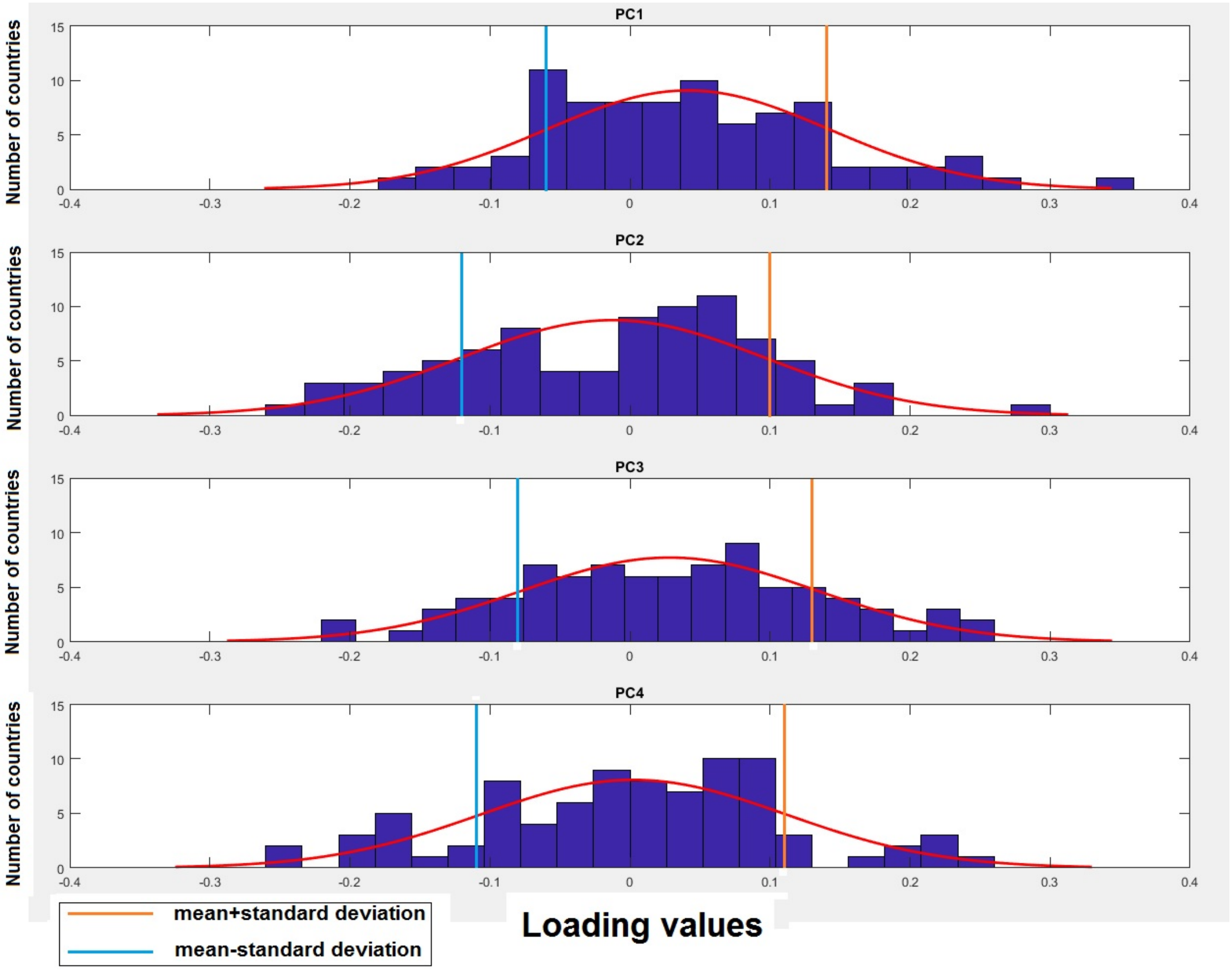
PC1
PC2
PC3
PC4
Number of countries
0
5
10
15
-0.4
-0.3
-0.2
-0.1
0
0.1
0.2
0.3
0.4
mean+standard deviation
mean-standard deviation
Loading values

Figure S7

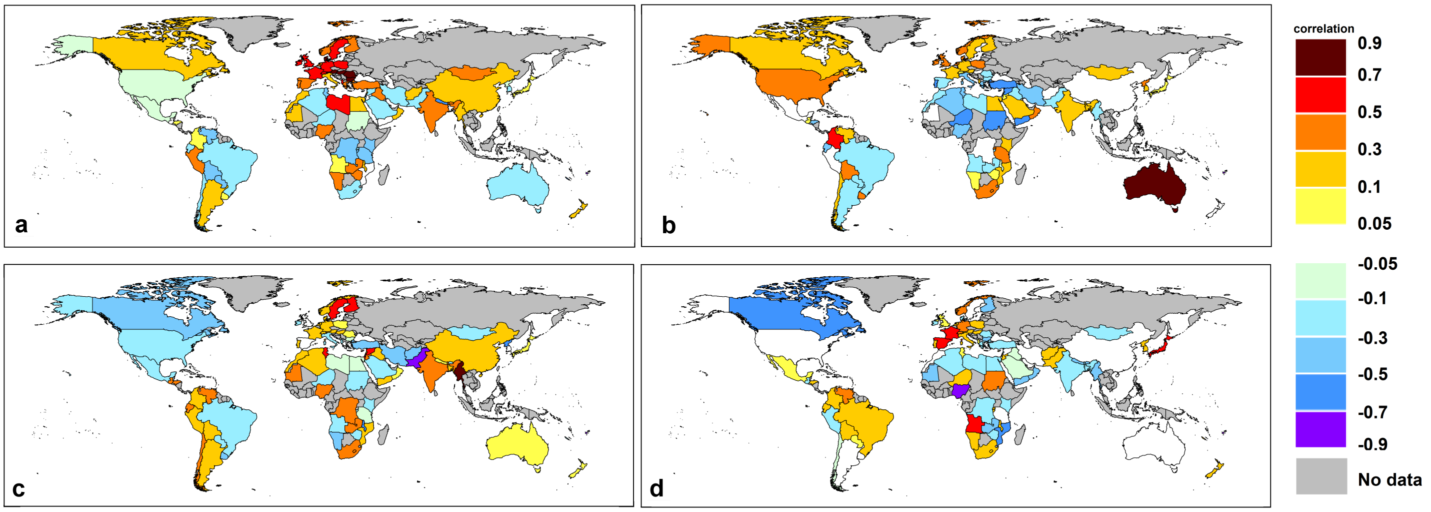

Figure S8

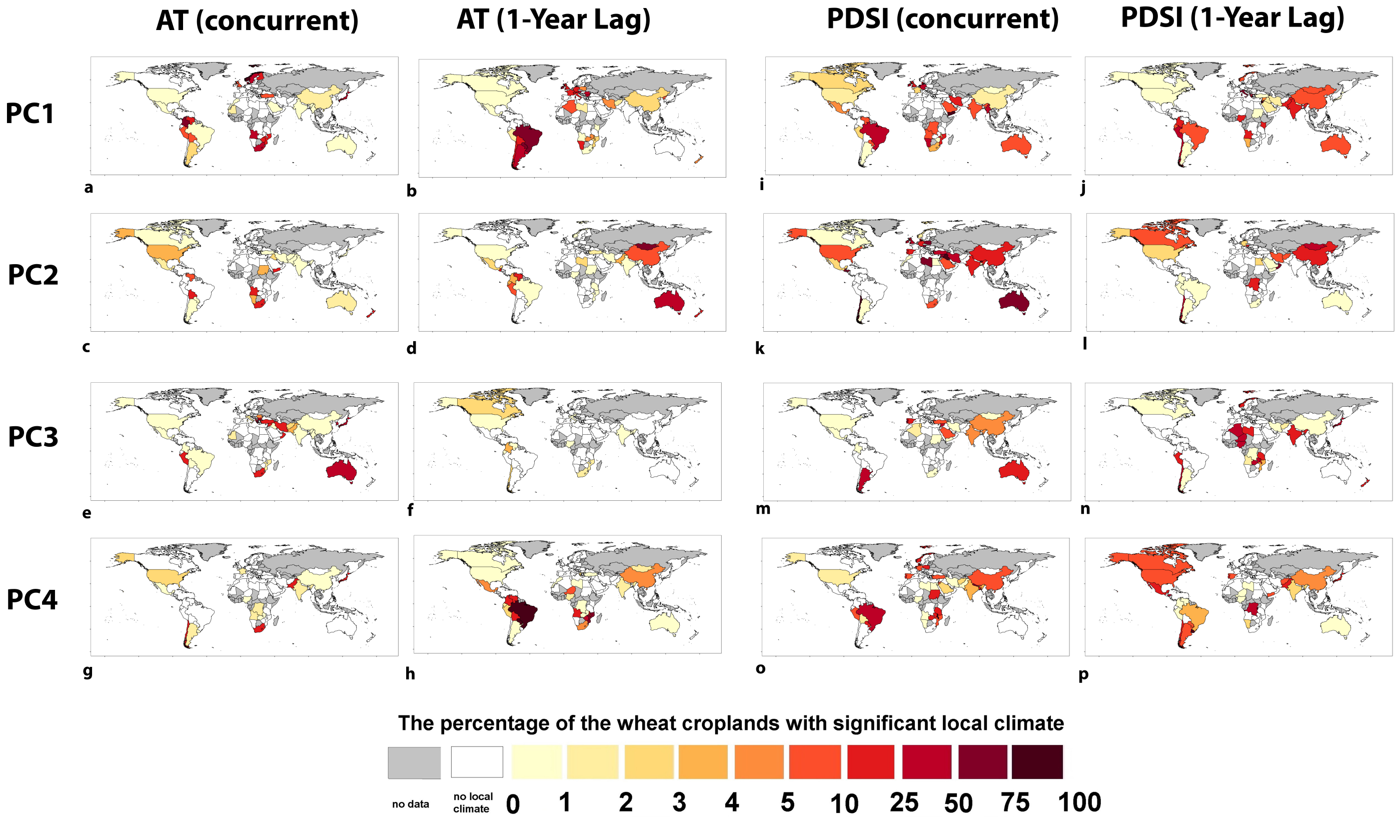

AT (concurrent)
AT (1-Year Lag)
PDSI (concurrent)
PDSI (1-Year Lag)
PC1
PC2
PC3
PC4
a
b
i
j
c
d
k
l
e
f
m
n
g
h
o
p
The percentage of the wheat croplands with significant local climate
no data
no local climate
0
1
2
3
4
5
10
25
50
75
100

Figure S9

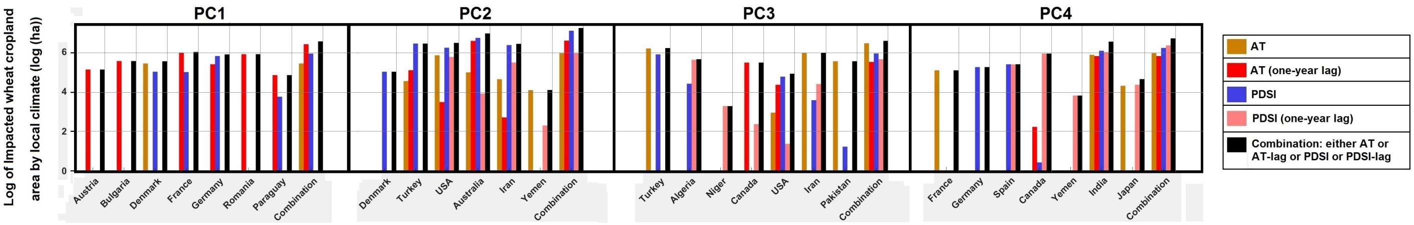

Log of Impacted wheat cropland area by local climate (log (ha))
PC1
PC2
PC3
PC4
0
2
4
6
Austria
Bulgaria
Denmark
France
Germany
Romania
Paraguay
Combination
Denmark
Turkey
USA
Australia
Iran
Yemen
Combination
Turkey
Algeria
Niger
Canada
USA
Iran
Pakistan
Combination
France
Germany
Spain
Canada
Yemen
India
Japan
Combination
AT
AT (one-year lag)
PDSI
PDSI (one-year lag)
Combination: either AT or AT-lag or PDSI or PDSI-lag

Figure S10

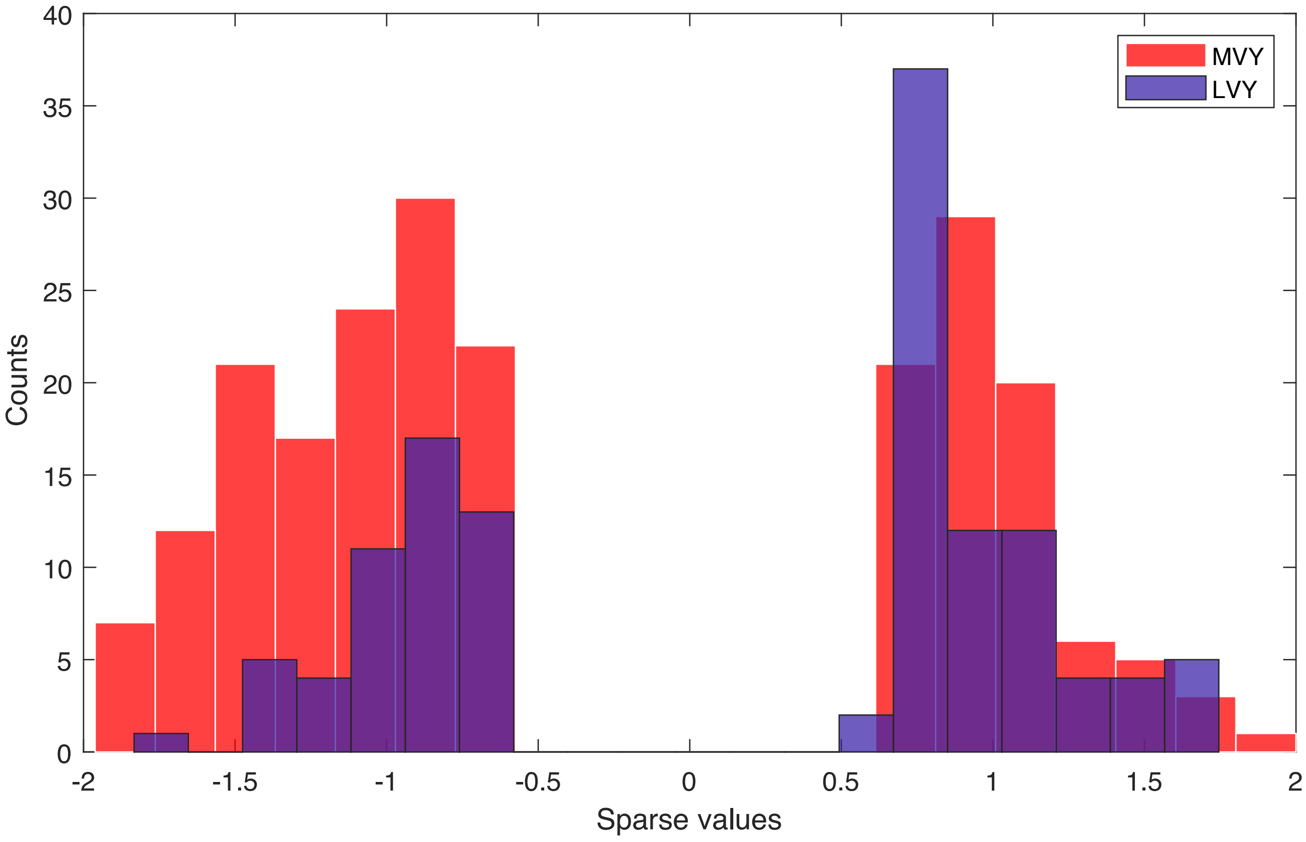

MVY
LVY
Counts
Sparse values
40
35
30
25
20
15
10
5
0
-2
-1.5
-1
-0.5
0
0.5
1
1.5
2

Figure S11

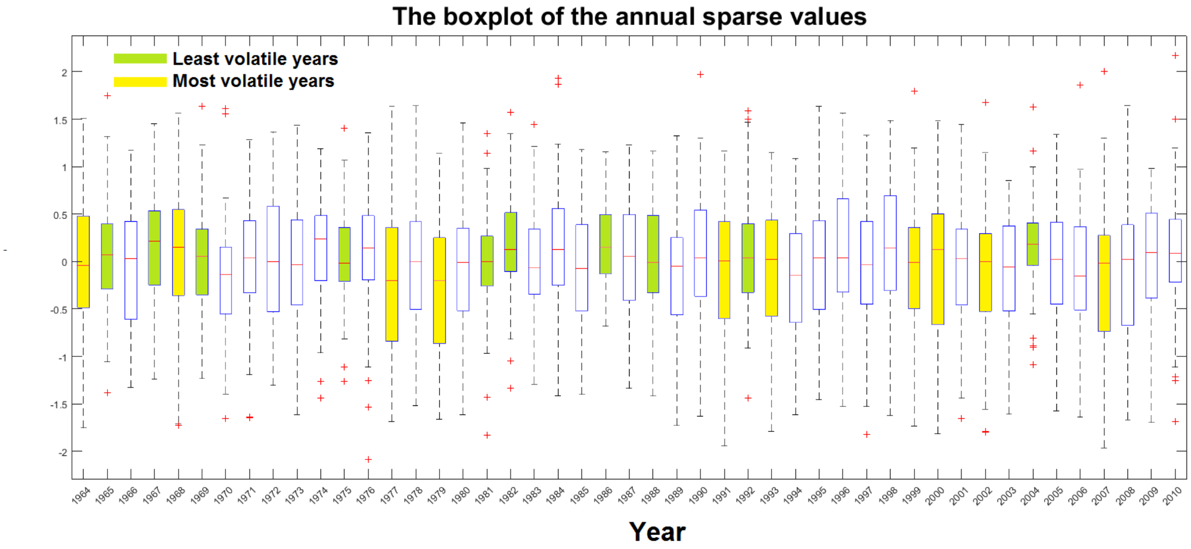
The boxplot of the annual sparse values
Least volatile years
Most volatile years
2
1.5
1
0.5
0
-0.5
-1
-1.5
-2
1964
1965
1966
1967
1968
1969
1970
1971
1972
1973
1974
1975
1976
1977
1978
1979
1980
1981
1982
1983
1984
1985
1986
1987
1988
1989
1990
1991
1992
1993
1994
1995
1996
1997
1998
1999
2000
2001
2002
2003
2004
2005
2006
2007
2008
2009
2010
Year

Figure S12

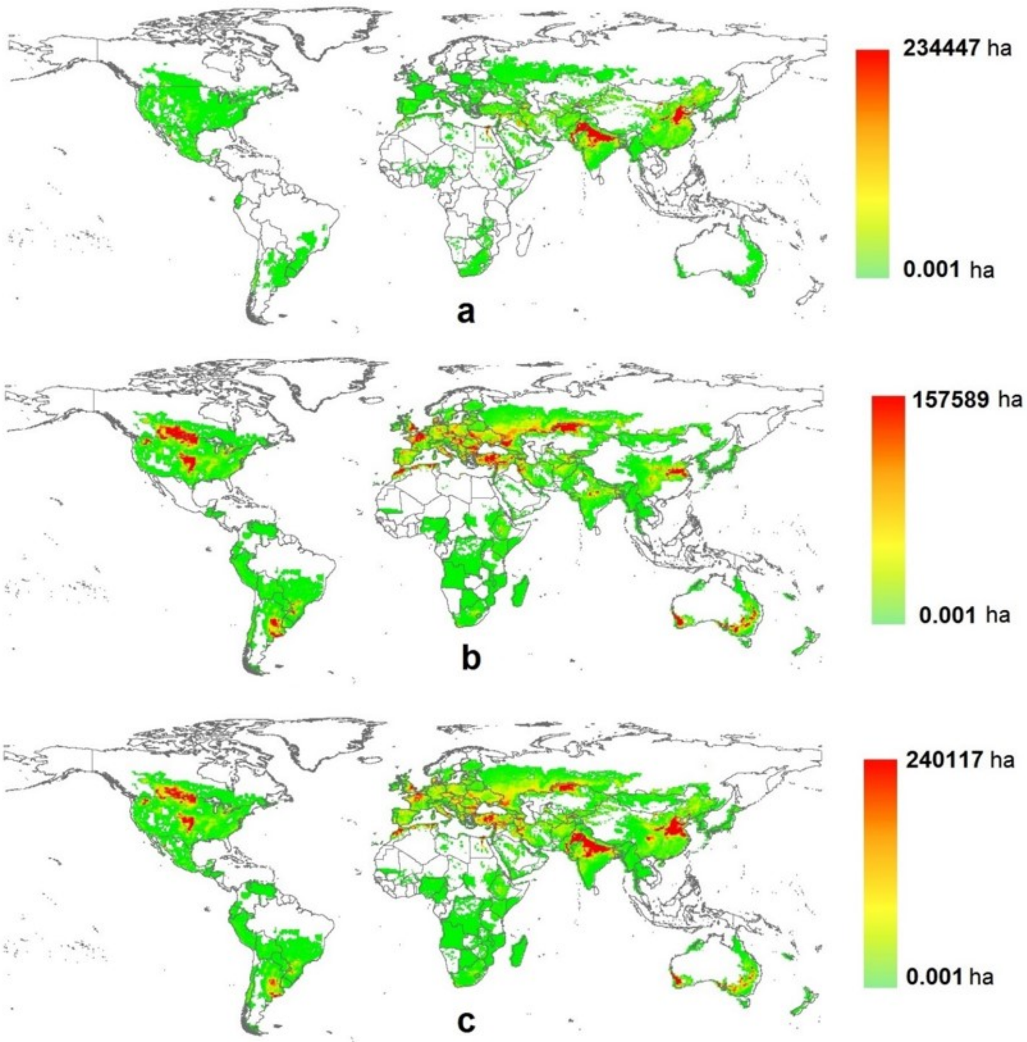

Figure S13

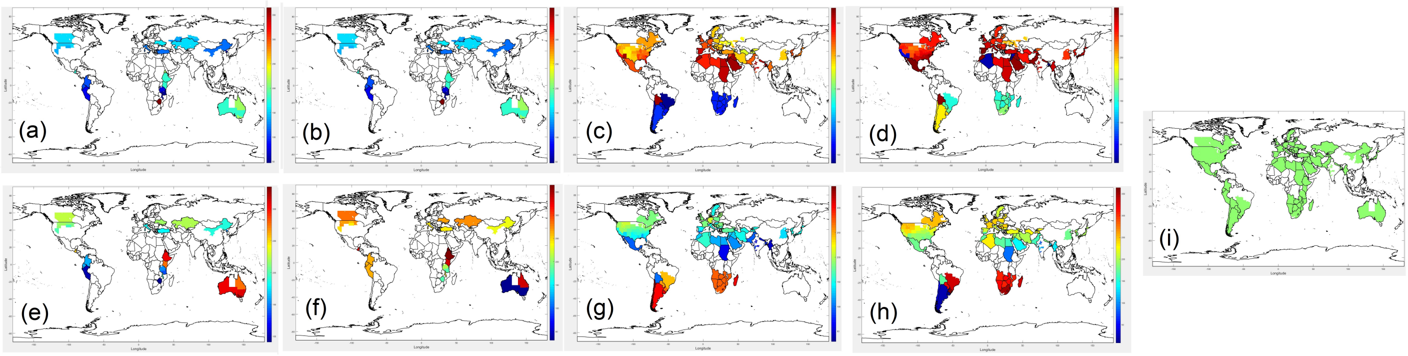

Supplement: Supplementary file 1 — Supplementary information. [file 41598_2020_60848_MOESM1_ESM.pdf]
